# SCTC: inference of developmental potential from single-cell transcriptional complexity

**DOI:** 10.1093/nar/gkae340

**Published:** 2024-05-06

**Authors:** Hai Lin, Huan Hu, Zhen Feng, Fei Xu, Jie Lyu, Xiang Li, Liyu Liu, Gen Yang, Jianwei Shuai

**Affiliations:** Wenzhou Key Laboratory of Biophysics, Wenzhou Institute, University of Chinese Academy of Sciences, Wenzhou, Zhejiang 325001, China; Oujiang Laboratory (Zhejiang Lab for Regenerative Medicine, Vision and Brain Health), Wenzhou, Zhejiang 325001, China; Institute of Applied Genomics, Fuzhou University, Fuzhou 350108, China; First Affiliated Hospital of Wenzhou Medical University, Wenzhou Medical University, Wenzhou 325000, China; Department of Physics, Anhui Normal University, Wuhu, Anhui 241002, China; Wenzhou Key Laboratory of Biophysics, Wenzhou Institute, University of Chinese Academy of Sciences, Wenzhou, Zhejiang 325001, China; Oujiang Laboratory (Zhejiang Lab for Regenerative Medicine, Vision and Brain Health), Wenzhou, Zhejiang 325001, China; Department of Physics, College of Physical Science and Technology, Xiamen University, Xiamen 361005, China; Wenzhou Key Laboratory of Biophysics, Wenzhou Institute, University of Chinese Academy of Sciences, Wenzhou, Zhejiang 325001, China; Chongqing Key Laboratory of Soft Condensed Matter Physics and Smart Materials, College of Physics, Chongqing University, Chongqing 401331, China; Wenzhou Key Laboratory of Biophysics, Wenzhou Institute, University of Chinese Academy of Sciences, Wenzhou, Zhejiang 325001, China; State Key Laboratory of Nuclear Physics and Technology, School of Physics, Peking University, Beijing 100871, China; Wenzhou Key Laboratory of Biophysics, Wenzhou Institute, University of Chinese Academy of Sciences, Wenzhou, Zhejiang 325001, China; Oujiang Laboratory (Zhejiang Lab for Regenerative Medicine, Vision and Brain Health), Wenzhou, Zhejiang 325001, China

## Abstract

Inferring the developmental potential of single cells from scRNA-Seq data and reconstructing the pseudo-temporal path of cell development are fundamental but challenging tasks in single-cell analysis. Although single-cell transcriptional diversity (SCTD) measured by the number of expressed genes per cell has been widely used as a hallmark of developmental potential, it may lead to incorrect estimation of differentiation states in some cases where gene expression does not decrease monotonously during the development process. In this study, we propose a novel metric called single-cell transcriptional complexity (SCTC), which draws on insights from the economic complexity theory and takes into account the sophisticated structure information of scRNA-Seq count matrix. We show that SCTC characterizes developmental potential more accurately than SCTD, especially in the early stages of development where cells typically have lower diversity but higher complexity than those in the later stages. Based on the SCTC, we provide an unsupervised method for accurate, robust, and transferable inference of single-cell pseudotime. Our findings suggest that the complexity emerging from the interplay between cells and genes determines the developmental potential, providing new insights into the understanding of biological development from the perspective of complexity theory.

## Graphical Abstract

**Graphical Abstract:**
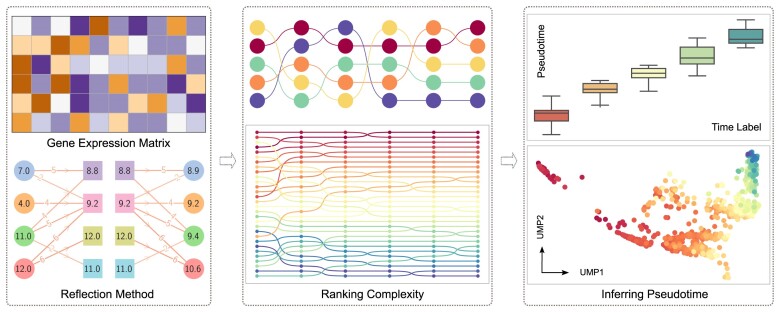

## Introduction

Single-cell RNA sequencing (scRNA-seq) technology (1–3) has emerged as a powerful tool for profiling gene expression in individual cells, providing unprecedented insights into the developmental process at single-cell resolution (4–6) . However, since scRNA-seq protocols result in cellular destruction, the technique can only obtain a snapshot of cells at the time of collection. This snapshot reflects a wide range of differentiation stages and cell states but lacks explicit temporal information (7). Hence, inferring the developmental potential of single cells from scRNA-Seq data and subsequently reconstructing the pseudotime of cell development is a fundamental and challenging task in the field of single-cell research (8–12). In the context of this task, a significant finding (13) has shown that the single-cell transcriptional diversity (SCTD), which is measured by the number of expressed genes per cell, can serve as a hallmark of cellular developmental potential. Specifically, the expression level of genes in individual cells generally decreases as cells undergo progressive differentiation, thereby providing a theoretical basis for developing a computational framework known as CytoTRACE based on scRNA-seq data to predict single-cell developmental potential (13). CytoTRACE has been widely used to infer the differentiation state of cells and has shown good performance in most datasets (13–16). Nonetheless, it has been reported that gene expression levels may not always monotonically decrease during development, particularly in the earliest stages where cells may exhibit a lower number of expressed genes compared to later stages (17). In such cases, we have found that the results obtained from gene diversity-based method like CytoTRACE cannot reliably reflect the true developmental potential and may lead to inaccurate estimations of single-cell pseudotime. This limitation may stem from the fact that gene diversity is solely based on the number of expressed genes, overlooking more subtle structural properties of gene expression, such as gene ubiquity, which refers to the number of cells that express a given gene. Consequently, a more sophisticated metric that accounts for these nuanced features may be more suitable for characterizing the developmental potential of single cells.

This issue is reminiscent of economic complexity theory, which provides a powerful framework for constructing predictors of a location’s developmental potential (18–21). Specifically, in the context of global trade, a country’s export diversity, measured by the number of products it exports, can only partially reflect its level of developmental potential, since some countries with the same number of exported products have significantly different developmental potential due to differences in the technical sophistication of their products. To account for more nuanced structural properties beyond the diversity of country export baskets, a novel pair of measures known as the Economic Complexity Index (ECI) and Product Complexity Index (PCI), have been proposed (18,19). These measures have been successfully applied to characterize a country’s level of development and developmental potential, and to explain cross-country variations in economic growth, providing important insights into patterns of economic development.

Drawing on the analogy of economic complexity theory, we introduce the concept of single-cell transcriptional complexity (SCTC) to quantify the complexity of gene expression patterns in individual cells. In this analogy, we view a cell as analogous to a country, and the genes expressed by a cell as analogous to the products exported by a country. We define the 0th-order complexity of a cell as its diversity, i.e., the number of genes expressed by that cell, and the 0th-order complexity of a gene as its ubiquity, i.e., the number of cells that express that gene. By interpreting scRNA-seq data as a bipartite network in which cells are connected to the genes they express, we can define higher-order complexities of cells and genes by correcting low-order complexities with more sophisticated network structure information. Additionally, we introduce two measures, namely the Cell Complexity Index (CCI) and Gene Complexity Index (GCI), to quantify the SCTC, as has been done in economic complexity theory. We demonstrate that the high-order complexities and CCI are more predictive of a cell’s developmental potential than the 0th-order complexity, particularly during the early stages of development when cells exhibit low gene expression but high complexity. Our study indicates that the complexity emerging from the interplay of cells and genes governs the developmental potential of cells, offering a novel framework for reconstructing the pseudotime of cell development and providing a new perspective from the viewpoint of complexity theory to understand biological development.

## Materials and methods

### Data preparation

We computed the SCTC metric on four scRNA-seq datasets. The first dataset, named Human Neuron Differentiation (HND) (17), was collected at 0, 1, 5, 7, 10 and 30 days during human neuron differentiation. We filtered the dataset by removing cells with more than 15% mitochondrial gene expression and excluding mitochondrial genes, resulting in a refined dataset containing 604 cells and 13 771 genes. The second dataset, referred to as Zebrafish Embryonic Cells (ZEB) (22), comprises 63 530 cells and 30 667 genes obtained at seven time points (4, 6, 8, 10, 16, 18 and 24 hours post-fertilization) during zebrafish embryonic development. The third and fourth datasets, Human Spermatogenesis (HSG) and Macaque Spermatogenesis (MSG), were extracted from the original scRNA-seq datasets of human and macaque testes (23), and include data of four stages of spermatogenesis: spermatogonia (denoted as stage 0), spermatocyte (stage 1), round spermatid (stage 2), and elongating spermatid (stage 3). The HSG dataset comprises 10 115 cells and 45 159 genes, and the MSG dataset comprises 19 467 cells and 22 863 genes. The selection criteria of these four datasets were based on the observation that the brain, testis and embryonic tissues are highly enriched in tissue-specific genes (24), which are known to play important roles in cellular differentiation and development (25).

The four datasets underwent a three-step preprocessing approach using the Python package Scanpy (26,27). First, we filtered out cells that had no gene expression and discarded genes that were not expressed in any cells. Next, we normalized the gene expression values using the Scanpy function ‘pp.normalize_total’. Finally, we applied a log_2_ transformation to the normalized data using the Scanpy function ‘pp.log1p’ to make the data more suitable for downstream analysis.

### Methodology

Inspired by economic complexity theory (18–20), we propose a method to quantify the complexity of cells and genes based on scRNA-seq data, and then infer the developmental potential of cells based on their complexity. Our method originates from two fundamental concepts: cell diversity (*k*_*c*, 0_) and gene ubiquity (*k*_*g*, 0_). Cell diversity is quantified as the sum of its gene expression levels across all genes, while gene ubiquity is calculated by the sum of its expression across all cells:


(1) \begin{eqnarray*} k_{c, 0}=\sum _{g} M_{cg} \end{eqnarray*}

(2) \begin{eqnarray*} k_{g, 0}=\sum _{c} M_{cg} \end{eqnarray*}

where *M*_*cg*_ is the element of the gene expression matrix, representing the expression level of gene *g* in cell *c*.

The scRNA-seq gene expression matrix can be conceptualized as a bipartite network, where cells and genes represent two types of nodes. Edges in this network indicate gene expression in cells, with the edge weights denoting expression levels. Therefore, *k*_*c*, 0_ and *k*_*g*, 0_ correspond to the degrees of cell and gene nodes, respectively. They serve as the initial metrics for quantifying complexity, which we define as 0th-order complexity. 0th-order complexity measures the connectivity of nodes solely based on the number of connections, ignoring the heterogeneity of connections. For example, consider two cells with the same total expression level. If one cell expresses a large number of high-complexity genes and the other expresses low-complexity genes, 0th-order complexity will fail to capture the difference in their complexity.

To overcome this limitation, it is necessary to adjust cell complexity based on gene complexity, and vice versa. This leads to a recursive relationship: a cell has high complexity if the genes it expresses have high complexity, and a gene has high complexity when the cells that express it have high complexity. This recursive relationship can be formalized into two equations, which are known as the reflection method in the literature of economic complexity theory (18,20):


(3) \begin{eqnarray*} k_{c, N}=\frac{1}{k_{c, 0}}\sum _{g} M_{cg} k_{g, N-1} \end{eqnarray*}

(4) \begin{eqnarray*} k_{g, N}=\frac{1}{k_{g, 0}}\sum _{c} M_{cg} k_{c, N-1} \end{eqnarray*}

where *k*_*c*, *N*_ and *k*_*g*, *N*_ represent the *N*th-order complexity of the cell *c* and the gene *g*, respectively. Equation (3) indicates that the *N*th order complexity of a cell is determined by the average (*N* − 1)th order complexity of the genes it expresses, and vice versa for gene complexity.

Based on Equations (3) and (4), we iteratively compute higher-order complexities starting from 0th-order complexity through recursive adjustments. This recursive process progressively incorporates information not covered by lower-order complexity. For example, the 0th-order complexity of a cell is only related to the number of genes it connects to, reflecting only the structural information of its nearest neighbors. In contrast, the first-order complexity considers which genes the cell connects to and which cells the connected genes connect to, integrating structural information from both nearest and next-nearest neighbors. Therefore, higher-order complexity integrates structural information from a wider range of the network, capturing the topological features of cells and genes in their connectivity network more accurately.

**Figure 1.**
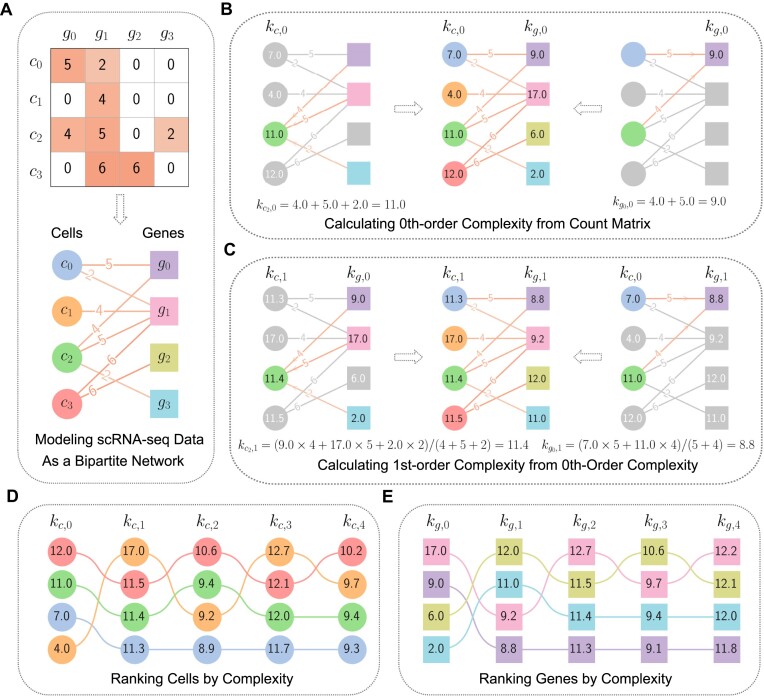
Toy model of single-cell transcriptional complexity. (**A**) The scRNA-seq count matrix can be represented as an adjacency matrix of a bipartite network, where gene expressions serve as edge weights connecting cells and genes. (**B**) Calculating 0th-order complexities of cells and genes by summing the weights of the edges connecting them. Examples are shown for cell *c*_2_ and gene *g*_0_. (**C**) Calculating 1st-order complexities of cells and genes by averaging over the the 0th-order complexities, weighted by the edge weights of the bipartite network, illustrated by the examples of cell *c*_2_ and gene *g*_0_. (**D**) Ranking cells by their complexity at different orders *N*. (**E**) Ranking genes by their complexity at different orders *N*.

To illustrate the SCTC calculation process in a clear and concise way, we introduce a toy model in Figure 1. This model consists of four cells expressing four genes, forming a bipartite network where gene expression levels act as edge weights connecting cells and genes (Figure 1A). Based on this network, we can recursively compute each order of complexity. Figure 1B shows how the 0th-order complexity for a cell or gene is calculated by simply summing the weights of its connected links. Higher-order cell complexity is then obtained by averaging the previous order’s gene complexity, weighted by the edge weights. Conversely, gene complexity is obtained by averaging the previous order’s cell complexity, again weighted by the edge weights. Figure 1C demonstrates this process for calculating first-order complexity based on 0th-order complexity. Finally, cells and genes are ranked based on their complexities at each order, as shown in Figures 1D and E. This ranking information then enables downstream analyses, such as inferring cell pseudotime.

The recursive method allows us to compute the complexity for each order. However, we have found that the complexity of cells (or genes) converges to a constant value once the order *N* exceeds a specific threshold *N*_*th*_. This convergence poses challenges in selecting an optimal order *N* that accurately represents the developmental potential of cells. An alternative approach, as demonstrated in studies on economic complexity (19,20), is to find an analytical solution to Equations (1-4).

Substitute (4) to (3) to obtain


(5) \begin{eqnarray*} k_{c, N}=\frac{1}{k_{c, 0}}\sum _{g} M_{cg} \frac{1}{k_{g, 0}}\sum _{c^{\prime }} M_{c^{\prime }g} k_{c^{\prime }, N-2} \end{eqnarray*}

which can be rewritten as:


(6) \begin{eqnarray*} k_{c, N}=\sum _{c^{\prime }} \widetilde{M}_{cc^{\prime }} k_{c^{\prime }, N-2} \end{eqnarray*}

where


(7) \begin{eqnarray*} \widetilde{M}_{cc^{\prime }}=\sum _{g}\frac{M_{cg}M_{c^{\prime }g}}{k_{c,0}k_{g,0}} \end{eqnarray*}

Equation (6) has a trivial solution, *k*_*c*, *N*_ = *k*_*c*, *N* − 2_ = 1, which corresponds to the eigenvector of matrix $\widetilde{M}{cc^{\prime }}$ associated with its largest eigenvalue. This eigenvector, with all entries being identical, does not provide any meaningful information and is thus ignored. We consider the eigenvector associated with the second-largest eigenvalue as the principal metric of cell complexity, denoted as $\overrightarrow{K}$. Each entry of $\overrightarrow{K}$ represents the complexity of a cell. However, $-\overrightarrow{K}$ is also an eigenvector of $\widetilde{M}{cc^{\prime }}$ corresponding to the same eigenvalue but in the opposite direction to $\overrightarrow{K}$. To choose the correct eigenvector, we utilize information from cell diversity. Since cell diversity is positively correlated with cell developmental potential, we calculate the Spearman Correlation Coefficient (SCC) between the cell diversity vector $\overrightarrow{K_{c, 0}}$=($k_{c_{0},0}$, $k_{c_{1},0}$, $k_{c_{2},0}$, …) and each of $\overrightarrow{K}$ and $-\overrightarrow{K}$ separately. The eigenvector with a positive SCC value is selected as the measure of cell complexity. Without loss of generality, assuming $\overrightarrow{K}$ has a positive SCC, we define CCI via normalizing $\overrightarrow{K}$:


(8) \begin{eqnarray*} CCI=\frac{\overrightarrow{K}-min(\overrightarrow{K})}{max(\overrightarrow{K})-min(\overrightarrow{K})} \end{eqnarray*}

where $max(\overrightarrow{K})$ and $min(\overrightarrow{K})$ are the maximum and minimum components of $\overrightarrow{K}$, respectively.

Gene Complexity Index (GCI) can be defined analogously to Equation (7) by swapping cell and gene indices. However, to avoid ambiguities arising from directionality concerns similar to the $\overrightarrow{K}$/ $-\overrightarrow{K}$ choice, we instead calculated GCI based on CCI as described in Equation (4):


(9) \begin{eqnarray*} Q_{g}=\frac{1}{k_{g, 0}}\sum _{c}M_{cg}CCI_{c} \end{eqnarray*}

Then we defined the normalized $\overrightarrow{Q}$ as GCI:


(10) \begin{eqnarray*} GCI=\frac{\overrightarrow{Q}-min(\overrightarrow{Q})}{max(\overrightarrow{Q})-min(\overrightarrow{Q})} \end{eqnarray*}

where $max(\overrightarrow{Q})$ and $min(\overrightarrow{Q})$ are the maximum and minimum components of $\overrightarrow{Q}$, respectively.

In summary, two methods are available for computing single-cell transcriptomic complexity through the scRNA-seq expression matrix. The first is a recursive iterative method that calculates the complexity of cells and genes from the 0th to *N*th order. The steps are as follows:

- Compute *k*_*c*, 0_ and *k*_*g*, 0_ using Equations (1) and (2).
- Calculate *k*_*c*, 1_ and *k*_*g*, 1_ using Equations (3) and (4).
- For *N*= 2, 3, ..., repeat step 2 to obtain the complexity of each order.
- Normalize the complexity obtained from each order.

The second method is an analytical approach, which involves:

- Construct the matrix $\widetilde{M}{cc^{\prime }}$ using Equation (7).
- Calculate the eigenvector $\overrightarrow{K}$ corresponding to the second-largest eigenvalue of $\widetilde{M}{cc^{\prime }}$.
- Compute cell diversity vector $\overrightarrow{K_{c, 0}}$, and calculate SCC between $\overrightarrow{K}$ and $\overrightarrow{K_{c, 0}}$. If SCC<0, set $\overrightarrow{K}$=$-\overrightarrow{K}$.
- Normalize $\overrightarrow{K}$ to obtain the Cell Complexity Index (CCI).
- Compute $\overrightarrow{Q}$ using Equation (9).
- Normalize $\overrightarrow{Q}$ to obtain the Gene Complexity Index (GCI).

Both methods offer insights into the intricacies of cells and genes. In this study, we specifically employ the recursive method to evaluate the efficacy of low-order and high-order complexity in deducing cell developmental potential. In most other scenarios, we utilize the analytically derived CCI and GCI as benchmarks for estimating cell developmental potential and inferring pseudotime.

## Results

### Comparison of pseudotime inferred by SCTD and SCTC

We compared the performance of SCTD and SCTC methods in pseudotime inference using four scRNA-seq datasets (HND, ZEB, HSG, and MSG). For each dataset, we computed the following metrics: normalized cell gene diversity, CytoTRACE pseudotime based on SCTD, and CCI pseudotime based on SCTC.

Cell gene diversity refers to the number of expressed genes in a cell. CytoTRACE operates under the assumption that cell diversity reflects developmental potential. Initially, it identifies genes whose expression levels highly correlate with the overall cell gene count. These selected genes are then used to compute the average expression level in each cell, known as the gene count signature (GCS). Next, CytoTRACE calculates and smoothes the GCS for each cell. Finally, the smoothed GCS is transformed into ranks, providing an indication of cell potency and allowing inference of cell pseudotime (13,28).

We employed the ‘CytotraceKernel’ function from the Python package CellRank (29) to compute the CytoTRACE pseudotime. Additionally, we utilized the CytoTRACE R package v0.3.310 (13) for pseudotime calculations and compared the results with those obtained using CellRank. Although there were minor discrepancies between the two software tools, these variations did not impact the conclusions drawn in our study (Supplementary Figure S1).

In the SCTC method, we utilize the *CCI*_*c*_ defined in Equation (8) as the measure of cell *c*’s developmental potential, and we define the pseudotime of the cell *c* as 1 − *CCI*_*c*_. This transformation ensures a negative correlation between CCI and developmental time, since higher CCI values correspond to earlier stages of development.

It’s noteworthy that both our method and CytoTRACE produced less than 1% ties in the results. Due to the small number of duplicates compared to the total number of time labels, their influence on the final outcomes is negligible. Consequently, we did not implement any additional processing to address these ties. For tied rankings, we maintained their original order in the input data, which aligns with the default practice in other pseudotime inference algorithms (28).

**Figure 2.**
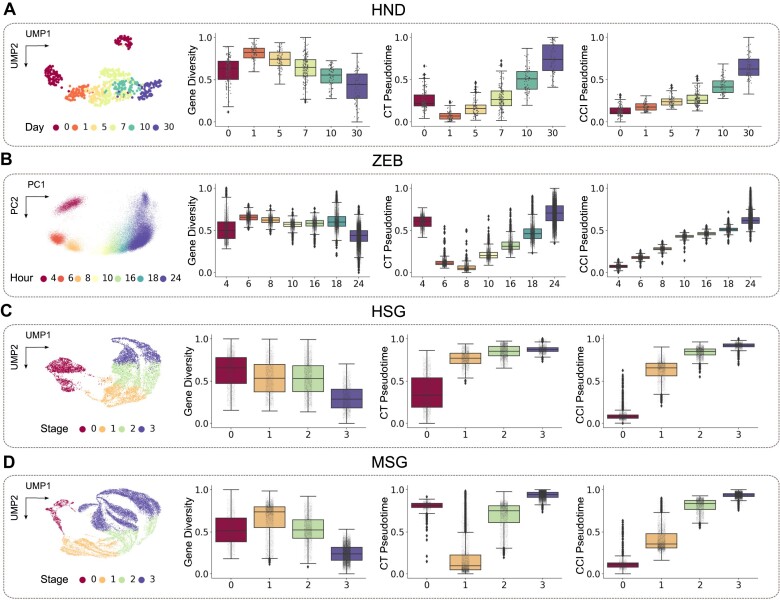
Comparison of CytoTRACE (CT) and CCI pseudotime for scRNA-seq data from (**A**) human neuron differentiation (HND), (**B**) zebrafish embryonic cells (ZEB), (**C**) human spermatogenesis (HSG) and (**D**) macaque spermatogenesis (MSG). For each dataset, UMAP or PCA plots of time point labels are presented, along with box plots showing normalized gene diversity, CT pseudotime and CCI pseudotime at each time point.

The results are presented in Figure 2 and Supplementary Figures S2. We observe that in the HND, ZEB, and MSG datasets, cells in the early developmental stages expressed fewer genes than those in the later stages (boxplots of gene diversity in Figure 2A, B and D). Consequently, the CytoTRACE pseudotime inference method, which is based on a negative correlation between cell developmental time and gene diversity, results in inaccurate estimations, particularly in the early stages. In contrast, the CCI pseudotime inference method, which is based on SCTC, offers a more reliable inference of pseudotime in the early developmental stages (Figure 2A, B and D).

Regarding the HSG dataset, we observed a monotonous decrease in the number of genes expressed by cells with the spermatogenesis process (Figure 2C). Hence, the inferred pseudotimes from both CytoTRACE and CCI align well with the temporal order of development. However, we found that the pseudotime distribution of the first stage of development inferred by CCI exhibited lower heterogeneity and greater separation from the second stage compared to the pseudotime inferred by CytoTRACE, suggesting that cell complexity metrics can more accurately distinguish the developmental potential of cells in the early stages. Notably, the pseudotime distributions inferred by gene diversity are significantly different between HCG and MCG cells, whereas those inferred by cell complexity are similar, indicating that SCTC can efficiently identify the same tissue-of-origin genetic characteristics in different species, rather than SCTD.

To evaluate the generalizability of the SCTC method, we additionally assessed its performance on 56 independent datasets. These diverse datasets included benchmarks used by CytoTRACE (13), Quasildr (a single-cell trajectory inference method) (12,30), and NCG (a single-cell pseudotime inference method) (31), as well as several other single-cell developmental studies (23,32,33). Compared to CytoTRACE on a total of 60 datasets, SCTC outperformed it on 39 (65%), indicating the advantage of SCTC method (*P*-value = 0.035). Details of the 60 datasets and the performance comparison are presented in Supplementary Table S1 and Supplementary Figure S3.

### Relationship between cell complexity and gene expression patterns in gene space

Taken together, our finding shows that SCTC is a more effective method for characterizing the developmental potential of cells and identifying their developmental stage in various cell environments, as compared to SCTD. This superiority may stem from SCTC’s ability to capture the more-sophisticated structural features of gene expression patterns within cells. Specifically, cells in early developmental stages require greater flexibility to have more opportunities to differentiate into various cell types and this flexibility is determined by the complexity of gene expression patterns within cells, rather than simply the overall expression levels of individual genes. This perspective finds its analogy in the economic complexity theory, where a country’s potential for development depends more on the complexity of its production structure rather than solely on the quantity of its products (18). This is because the complexity of a country’s production structure reflects its ability to access a wider range of resources and knowledge, which ultimately enhances its production capacity and provides more opportunities for future development (34).

In the context of economic complexity theory, the complexity of a country’s productive structure is reflected in the distribution of its products within the ‘product space’, a network of relationships among different products. Research has shown that countries with higher development potential tend to produce products that are located at the core of this network with stronger connectivity (34). This positioning advantage enables them to expand their production capabilities through numerous connections, thereby enhancing their potential for future growth. Inspired by this concept, we have introduced the idea of a ‘gene space’, allowing for a deeper exploration of the relationship between cell complexity and gene expression patterns from the perspective of gene-gene interactions.

To define the gene space, we draw an analogy between cells and countries, and between genes and products. Then we calculated the Revealed Comparative Advantage (RCA) (35) of gene *g* expressed by cell *c*:


(11) \begin{eqnarray*} RCA_{c,g}=\frac{x_{c, g}/\sum _{g}x_{c, g}}{\sum _{c}x_{c, g}/\sum _{c, g}x_{c, g}} \end{eqnarray*}

which measures whether a cell *c* expresses gene *g* more prominently, relative to its overall gene expression, compared to the ‘average’ cell. If *RCA*_*c*, *g*_ > 1, it means that the cell has a higher relative advantage in expressing that specific gene. Subsequently, the proximity ϕ between genes *i* and *j* is defined as the minimum value of the pairwise conditional probabilities of a cell expressing gene *i* significantly, given that it also expresses gene *j* significantly:


(12) \begin{eqnarray*} \phi _{i, j}=min{P(RCAx_{i}|RCAx_{j}), P(RCAx_{j}|RCAx_{i})} \end{eqnarray*}

where


(13) \begin{eqnarray*} P(RCAx_{i}|RCAx_{j})=\frac{\sum _{c}[RCA_{c, i} \ge 1 \& RCA_{c, j} \ge 1]}{\sum _{c}[RCA_{c, j} \ge 1]} \end{eqnarray*}

**Figure 3.**
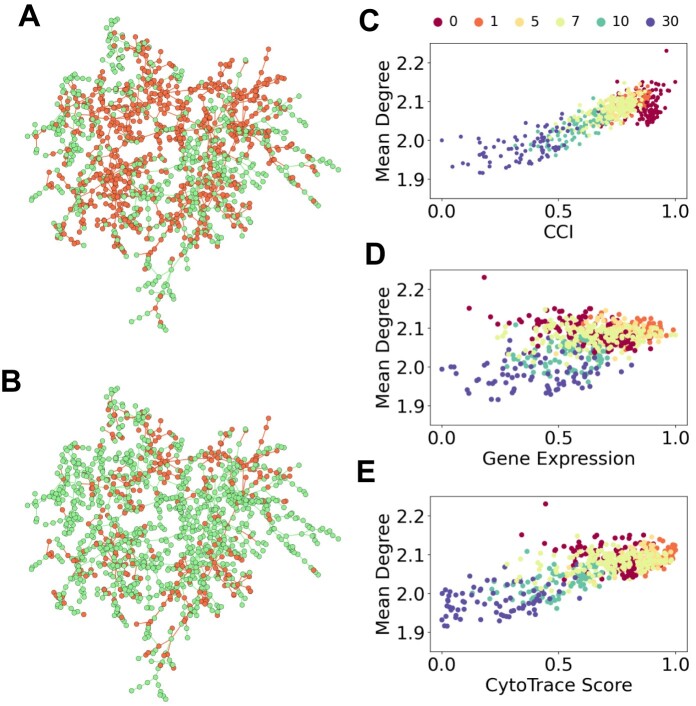
Relationship between cell complexity and gene expression patterns in gene space. (**A, B**) Gene expression profiles of cells with the highest (A) and lowest (B) complexity are mapped to the gene space, where each node represents a gene. Green nodes indicate genes not expressed in the cell, while red nodes signify expressed genes. (**D, E**) Scatter plots illustrating the correlations between the mean degree of genes expressed by cells and three cellular metrics: CCI (C, PCC=0.844), normalized gene expression level (D, PCC=0.390), and CytoTRACE score (E, PCC=0.733).

The proximity matrix, calculated using Equation (12), can be used as the adjacency matrix of the gene network. Moreover, we computed the maximum spanning tree of this network to represent the gene space, allowing us to simplify the network structure while preserving its critical connectivity patterns. We provide an example of the gene space in Figure 3A and B, where we randomly selected 10% of the genes from the HND dataset for visualization. By utilizing the scRNA-seq count matrix, we determined the cell complexity using Equation (8) and constructed the gene space using Equation (12). Afterwards, we mapped the gene expression profiles of cells with the highest complexity (Figure 3A) and lowest complexity (Figure 3B) onto the gene space. We observed that cells with higher complexity appeared to exhibit denser and more concentrated gene expression patterns in the gene space, whereas cells with lower complexity seemed to display sparser and more dispersed patterns.

To further confirm this observation quantitatively, we derived the gene space from the complete HND dataset and calculated the average degree of the genes expressed by each cell. Subsequently, we examined the Pearson Correlation Coefficients (PCC) between the average degree and three metrics: cell complexity measured by CCI, cell expression levels, and CytoTRACE scores. The analysis revealed a strong correlation between cellular complexity and average degree, as depicted in Figure 3C (PCC=0.844). Conversely, the correlation between cell expression levels and average degree was found to be considerably weaker (Figure 3D, PCC=0.390). Although the use of the CytoTRACE algorithm significantly improved the correlation compared to cell expression alone, it failed to capture the structural features of gene expression patterns at day 0 (Figure 3E, PCC=0.733).

These findings indicate that SCTC can effectively capture the complex structural information of the scRNA-seq count matrices, thereby providing a more accurate estimation of cell developmental potential. Specifically, SCTC integrates topological information of the network at a global level through iterative computation, reflecting the distribution pattern of cellular genes within the gene space. Cells of higher complexity tend to express genes clustered in the core region of the gene space. These core genes have a high degree of connectivity and interact more with other genes, thus they may play a crucial role in biological processes such as gene regulation. The complexity of these gene expression patterns potentially provides cells with the capability to adapt to diverse environmental demands. This adaptability offers broader possibilities for cellular diversification and differentiation, consequently leading to a higher developmental potential.

### Impact of complexity order on the inference of developmental potential

**Figure 4.**
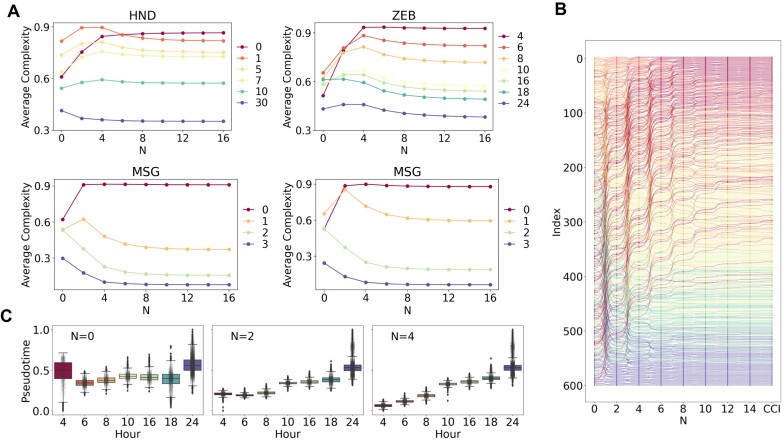
Cell complexity as a function of complexity order *N*. (**A**) Average cell complexity at different time points as a function of complexity order *N*. (**B**) 604 HND cells ranked by *N*th-order complexity and CCI. (**C**) Box plots of pseudotime of ZEB cells inferred by cell complexity with different order *N*.

To investigate the impact of complexity order on the inference of developmental potential, we employed recursive calculations based on Equations (1-4) (Figure 4) to determine the cell complexity as a function of complexity order *N*. As the even-order complexities of cells display negative correlations with the odd-order complexities (Supplementary Figure S4) (18), we focused on the even orders of cell complexity and the odd orders of gene complexity. Figure 4A depicts the relationship between the average cell complexity and the complexity order *N* at different time points across the four scRNA-seq datasets. Our analysis revealed that cell complexities at lower orders (*N* < 8 for HND, *N* < 4 for ZEB and MSG, and *N* < 2 for HSG) inadequately reflected the actual developmental stages and failed to accurately characterize the developmental potential of cells. With increasing complexity order *N*, the average cell complexity exhibits better alignment with the actual time points, indicating that higher-order complexities more accurately captured the developmental potential of cells. However, beyond a certain threshold (*N*_*th*_ = 16 for HND, *N*_*th*_ = 28 for ZEB, *N*_*th*_ = 52 for MSG, and *N*_*th*_ = 60 for HSG), further increases in the complexity order *N* caused the cell complexity to converge to the same value through recursion (Supplementary Figure S5).

To compare the results of numerical and analytical calculation methods, the 14th-order complexity was chosen to infer the pseudotime of cells in four datasets. As shown in Supplementary Figure S6, the pseudotime of the CCI obtained by analytical calculation is consistent with the numerical calculation results *k*_*c*,14_, indicating that the second eigenvector of Equation (7) accurately captures the information related to high-order cell complexity. Therefore, the CCI can serve as a criterion for selecting an appropriate value of *N* when applying *N*th-order complexity. To calculate the correlation (SCC) between each order of complexity and the CCI, the *N* value corresponding to a sufficiently large SCC, or the *N* value with the highest SCC within a broader range, can both serve as appropriate criteria of complexity (Supplementary Figure S7).

Figure 4B presents the ranking results of the 604 HND cells based on their *N*th-order complexity, where the last column represents the rankings obtained using CCI. The color scheme used follows that of the HND data in Figure 4A and corresponds to different time points. It is clear that as the complexity order *N* increases, the cells undergo a continuous rearrangement process, eventually reaching a stable state that aligns with the rankings obtained using CCI. This trend indicates that higher-order complexities result in a more accurate ranking of cells based on their developmental progression.

In Figure 4C, we present the inferred pseudotime of ZEB cells using different orders of cell complexity (*N* = 0, 2 and 4, respectively). As expected, the inferred pseudotime corresponding to the 0th-order complexity is incorrect. However, after four rounds of recursion, the cells can be efficiently ranked using the fourth-order complexity (Figure 4C), demonstrating the effectiveness of higher-order complexities in capturing development-relevant information in cells.

Higher-order complexity plays a crucial role in accurately inferring the developmental potential of single cells due to its ability to encompass a wider range of information. Each high-order complexity, as determined by Equations (1-4), is obtained through the iterative calculation of the average value of previous-level properties of neighboring nodes using the method of reflections (34). This methodology allows us to integrate structural information from a bipartite network that includes a broader range of cells and genes, effectively correcting the information captured by lower-order complexities. This approach shares similarities with methods commonly employed in deep learning, which has been widely applied in single-cell research in recent years (36–38). Convolutional neural networks (CNNs) (39) and graph neural networks (GNNs) (40,41) are common models in deep learning that aggregate information from neighboring nodes to enhance feature extraction accuracy.

Due to the complexity and nonlinearity of model parameters, the aggregated information in deep learning models is often uninterpretable. In contrast, our model provides interpretable explanations for complexity metrics by explicitly defining multi-order measures. Table 1 presents the interpretations of the first three orders complexity of cell and gene (18), highlighting that high-order complexity integrates a broader range of information than low-order complexity. For example, *k*_*c*,1_ only considers the ubiquity of genes expressed by cell *c*, focusing on the expression profile information of a single cell, whereas *k*_*c*,2_ considers diversity of other cells with similar gene expression profiles to cell *c*, and characterizes cell *c* from a population perspective, thereby encompassing a wider range of information.

**Table 1.** Interpretation of the first three orders complexity of cell and gene

Definition	Description	Interpretation
*k*_*c*, 0_ (Diversity)	Number of genes expressed by cell *c*.	How many genes are expressed by cell *c*?
*k*_*g*, 0_ (Ubiquity)	Number of cells expressing gene *g*.	How many cells express gene *g*?
*k*_*c*, 1_	Average ubiquity of the genes expressed by cell *c*.	How common are the genes expressed by cell *c*?
*k*_*g*, 1_	Average diversity of the cells expressing gene *g*.	How diverse are the cells that express gene *g*?
*k*_*c*, 2_	Average diversity of cells with a gene expression profile similar to cell *c*.	How diverse are cells expressing genes similar to those of cell *c*?
*k*_*g*, 2_	Average ubiquity of the genes expressed by cells that express gene *g*.	How ubiquitous are the genes expressed by cells expressing gene *g*?

As the order of complexity increases, interpreting its meaning becomes increasingly challenging. However, in the field of economic complexity theory (18), analytically solving the recursion Equations (1-4) reveals that *N*th-order complexity captures the characteristics of nodes in the network by combining the properties of its neighbors, and the coefficients of the linear combination being the probability of a random walk reaching these neighboring nodes after *N* steps. This implies that a node located centrally within the network will have higher *N*th-order complexity. Through *N* steps of random walks, such a node can reach numerous neighbors with high complexity. Therefore, the *N*th-order complexity effectively measures the significance of nodes within a network.

As we previously observed gene expression patterns in the gene space (gene–gene network), cells or genes with high-order complexity are also found to be located at the core of the cell-gene interaction network. These cells or genes potentially play significant roles in biological development and exhibit higher developmental potential. The superior performance of higher-order complexity over lower-order complexity in inferring cell developmental potential underscores the fact that cellular development is more influenced by intricate interactions between cells and genes than solely by gene expression levels. This interpretation of complexity metrics enhances our understanding of the key factors determining cell developmental potential.

### Evaluating gene diversity and complexity for distinguishing developmental stages

Gene diversity was previously defined as the number of genes expressed by a cell. We now denote the average GCI of genes expressed by an individual cell as the gene complexity of the cell. To evaluate the discriminative ability of gene diversity and gene complexity in distinguishing different developmental stages, we conducted a comparative analysis of these measures in four datasets (HND, ZEB, HSG and MSG).

**Figure 5.**
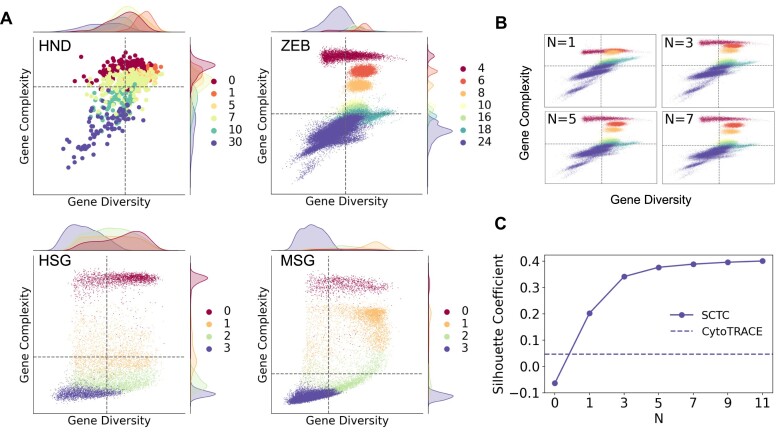
Evaluating gene diversity and complexity for distinguishing developmental stages. (**A**) The diversity-complexity diagrams of single-cell gene expression, with unique colors representing cells from different developmental stages. The dashed lines indicate the mean gene diversity and mean gene complexity averaged over all cells. (**B**) The diversity-complexity diagram as a function of gene complexity order *N* for the ZEB data. (**C**) Silhouette coefficient of gene complexity as a function of complexity order *N* for the ZEB data, where *N* = 0 represents the gene diversity. The dashed line indicates the Silhouette coefficient of CytoTRACE score.

Figure 5A illustrates the marginal distributions of gene diversity (X-axis) and gene complexity (Y-axis) for cells at each developmental stage. The marginal distribution of gene diversity exhibits a wide range of variability and overlapping regions, making it insufficient to differentiate developmental stages by gene diversity alone. In contrast, the marginal distribution of gene complexity displays pronounced separations, especially during the early stages of development. In these early stages, gene complexity values are notably higher compared to the later stages. This demonstrates the role of complexity metrics in effectively discerning stages of cell development, particularly in distinguishing the early developmental stages.

Figure 5A clearly demonstrates the significant heterogeneity in gene expression levels among cells during the early stages of development. A large number of cells in the first stage exhibit considerably lower expression levels compared to the subsequent stages. This pattern is particularly evident in the ZEB dataset (Figure 5A, top right). Remarkably, despite the lower expression levels during this stage, these cells display a high level of complexity, with complexity values falling within a relatively narrow range. This suggests that cells in the early developmental stages possess higher regulatory complexity and potential functional diversity, primarily determined by the structure of gene expression patterns rather than the overall gene expression levels.

In contrast, the majority of cells in the final stage of development are clustered within the third quadrant, as indicated by the two dashed lines (Figure 5A). This positioning signifies the presence of low levels of both gene diversity and complexity in these differentiated cells, suggesting a less intricate gene regulation relationship compared to earlier developmental stages.

We further investigated the joint distributions of gene diversity and complexity across different orders (*N* = 1, 3, 5, 7) using the ZEB dataset (Figure 5B). The results demonstrate that as the order of gene complexity increases, the ability to discriminate between different cell differentiation stages improves, particularly for early developmental stages. To quantitatively evaluate this observation, we calculated the Silhouette coefficient (42), a metric that assesses the distinctiveness and separation of clusters. This analysis was performed using the developmental stage labels in reverse temporal order and the average gene complexity of cells with orders *N* ranging from 1 to 11. Additionally, we computed the Silhouette coefficient for gene diversity (represented by *N* = 0) and the CytoTrace score for comparison purposes.

The results from the ZEB dataset are presented in Figure 4C. As shown, the discriminatory power of gene complexity at the first order surpasses that of gene diversity and CytoTRACE in distinguishing cell developmental stages. Furthermore, the Silhouette coefficient increases with higher complexity order *N*. Similar trends are observed in the other datasets (Supplementary Figure S8), providing further validation of the effective discrimination of cell developmental stages by higher-order complexity.

### Genes with different complexity are associated with specific developmental stages

In the CytoTRACE model, genes are ranked based on the correlation between their expression levels across cells and the CytoTRACE scores of those cells (13). In our model, leveraging the symmetry of the cell–gene bipartite network, we can computationally determine the multi-order complexity for both cells and genes using the reflection method (Equations (1)-(4)). Alternatively, the CCI (Equations (8)) and the GCI (Equations (10)) can be analytically derived. This enables ranking genes based on their inherent multi-order complexity measures or overall GCI values, contrasting with the correlation-based gene ranking approach used in CytoTRACE.

To evaluate the association between gene complexity and developmental stages, we identified marker genes for each stage in the scRNA-seq dataset using the Wilcoxon rank-sum test in Scanpy (26,43). For each stage, the top 10 genes with the most significant differential expression were selected. We then examined the relationship between the complexity-based ranking of these genes and their actual stage assignments.

**Figure 6.**
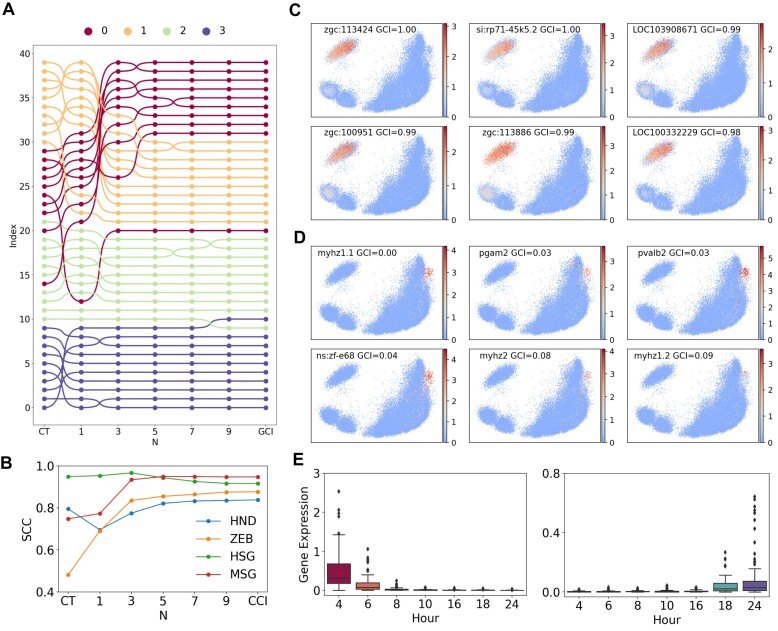
Genes with different complexity are associated with specific developmental stages. (**A**) Rankings of the marker genes identified for each developmental stage in the MSG dataset, based on CytoTRACE score, multi-order complexity (odd order *N* from 1 to 9), and Gene Complexity Index (GCI). (**B**) Spearman Correlation Coefficient (SCC) between gene rankings and actual developmental orderings across four datasets. Rankings are based on CytoTRACE score, multi-order complexity (odd order *N* from 1 to 9), and GCI. (**C**) The 6 genes with the highest complexity in the ZEB data exhibit preferential expression in the earliest developmental stage. (**D**) The six genes with the lowest complexity mainly express in the last developmental stage for ZEB data. (**E**) The highest 100 complexity genes in the ZEB data are selectively expressed in early development (left), while the lowest 100 complexity genes show specificity for later stages (right).

Figure 6A shows the gene rankings in the MSG dataset based on different complexity measures. The first and last columns correspond to the rankings by cytoTRACE and GCI, respectively. Notably, the rankings by cytoTRACE and first-order complexity exhibit an inverse relationship with the actual developmental stages for stages 0 and 1. However, at the third-order complexity, the rankings become consistent with the true developmental trajectory. During this transition, a considerable number of genes undergo significant ranking jumps, indicating that higher-order complexity integrates crucial topological information to align with developmental progression.

Similar trends were observed across the other datasets as well. In Figure 6B, we calculated SCC between the gene rankings and the actual temporal orderings using four datasets. Except for the HSG dataset, where different metrics yielded comparably high SCC values, the rankings based on higher-order complexity and CCI demonstrated closer agreement with the developmental trajectory than lower-order complexity and CytoTRACE in the other three datasets. This reveals that, by incorporating global topological characteristics, higher-order gene complexity provides a more intrinsic measure for revealing the developmental regulation and temporal expression patterns of genes.

To further investigate the association between gene complexity and developmental stages, we examined the stage distributions of genes ranked highest and lowest in terms of complexity. As illustrated in Figures 6C and D using the ZEB dataset, the 6 genes with the highest GCI preferentially express in the earliest developmental stage, whereas the six genes with the lowest GCI are primarily enriched in the final stage. This distinct distribution pattern was consistently observed across all four datasets (Supplementary Figure S9). Additionally, analyzing the expression of the highest 100 GCI genes and the lowest 100 GCI genes in each dataset revealed high-complexity genes are selectively expressed in early development, while low-complexity genes show specificity for later stages (Figure 6E and Supplementary Figure S10).

Collectively, these results demonstrate genes with different developmental stage specificities are inherently characterized by varying complexity levels. The complexity spectra provide a quantitative and intrinsic metric to map dynamic gene regulation across cell fate trajectories, which may help to uncover mechanisms controlling cell potency and lineage commitment.

### Transferability and robustness evaluation of the single-cell transcriptional complexity model

In our model, cell and gene complexity are defined recursively based on each other through Equations (3) and (4). This enables computing cell complexity from gene complexity, and vice versa. Moreover, many genes are shared across different single-cell datasets. Therefore, the gene complexity derived from one dataset can be utilized to calculate the cell complexity of another dataset, demonstrating the transferability of our model.

**Figure 7.**
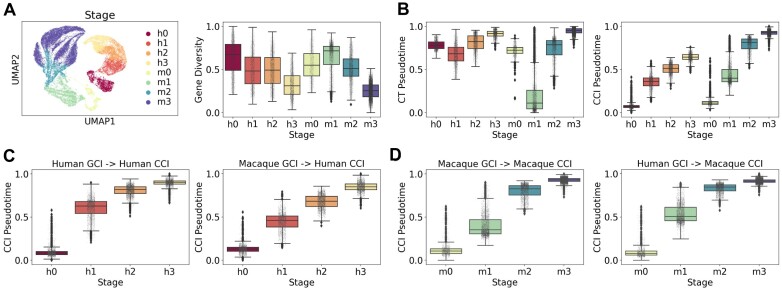
Transferability and robustness evaluation of the single-cell transcriptional complexity model. (**A**) UMAP plot and normalized gene diversity distribution of the merged dataset. “h” denotes Human and “m” denotes Macaque. (**B**) Box plots of CytoTRACE pseudotime (left) and CCI pseudotime (right) of the merged dataset. (**C**) Human CCI pseudotime computed by Human GCI (left) and by Macaque GCI (right). (**D**) Macaque CCI pseudotime computed by Macaque GCI (left) and by Human GCI (right).

To evaluate this, we first merged the HSG and MSG datasets into a mixed dataset containing 29 591 cells and 14 405 shared genes. The UMAP visualization and normalized gene diversity distribution of this combined dataset are shown in Figure 7A. We then computed the pseudotimes using CytoTRACE and SCTC method. As depicted in Figure 7B, SCTC maintained the correct temporal ordering of human and macaque cell types, while CytoTRACE showed more disordered results. This comparison highlights the higher robustness of our SCTC model on heterogeneous datasets.

Interestingly, we have observed that the CCI-inferred pseudotime distribution of human sperm cells is lower than that of macaque sperm cells (Figure 7B, right), implying that human sperm cells possess higher overall complexity. This phenomenon is even more pronounced when visualized through the diversity-complexity diagrams (Supplementary Figure S11), aligning with a previous hypothesis (25) that the increased complexity of humans is not only present at the phenotypic organismal level but also extends to the genomic and cellular levels. Our results provide single-cell resolution evidence supporting the view that human cells have more complex regulation at the transcriptional level compared to other primate species.

We then divided the mixed dataset into new HCG and MCG datasets containing only human and macaque cells, but sharing all 14 405 genes. Next, we calculated GCI for each dataset separately. Leveraging Equation (3), the human and macaque GCIs were utilized to derive human CCI pseudotime (Figure 7C), and vice versa for macaque CCI pseudotime (Figure 7D). The high consistency between CCI pseudotimes calculated using GCIs from the same versus different datasets demonstrates the transferability of our complexity model. Consequently, the conserved gene complexity ranking across species suggests new insights into extending developmental potential inference across different model organisms using the complexity theory framework.

### Stability and robustness of SCTC to dropouts and imputations

Dropout is a common phenomenon observed in scRNA-Seq datasets (44). It refers to missing values in sequencing data due to technical limitations. To assess its impact on the SCTC method and compare it with the CytoTRACE, we simulated dropout events at varying dropout rates (e.g., 0.1–0.9) on four datasets. Specifically, elements of the scRNA-seq count matrix were randomly set to zero according to the specified dropout rate. We then evaluated the performance of both methods in inferring cell pseudotime under these dropout conditions, quantifying their accuracy using SCC. For each dataset, ten dropout simulations were conducted, and the results were averaged to assess algorithm stability.

**Figure 8.**
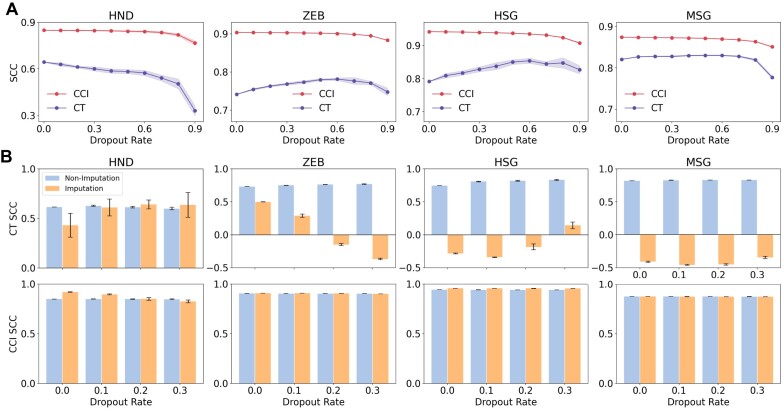
Stability and robustness of SCTC methods to dropouts and imputations. (**A**) Comparative accuracy of pseudotime inference using CytoTRACE and SCTC methods across four datasets (HND, ZEB, HSG and MSG) subjected to dropout simulations at different dropout rates ranging from 0.1 to 0.9. Dotted lines represent the mean results obtained from ten sampling calculations, while shaded regions illustrate the standard deviation across ten dropout simulations. (**B**) The performance of CytoTRACE and SCTC was evaluated on four datasets both without (blue) and with imputation (orange). These datasets were subjected to dropout simulations before imputation with dropout rates of 0.0, 0.1, 0.2 and 0.3, respectively. The error bars represent the standard deviation across ten sampling experiments.

Figure 8A compares the performance of CytoTRACE and SCTC methods in inferring cell pseudotime under different dropout rates. Across multiple datasets, the CytoTRACE method exhibits significant uncertainty in response to dropout events. Specifically, in the HND and MSG datasets, its accuracy notably declines when dropout rates reach 0.9. However, for the ZEB and HSG datasets, increasing the dropout rate shows a slight improvement in accuracy. Moreover, CytoTRACE shows larger variations in results across repeated computations on the same dataset, compared to SCTC.

In contrast, the SCTC method shows a slight decrease in accuracy as the dropout rate increases, but it still significantly outperforms CytoTRACE overall. Moreover, the relationship between accuracy and dropout rate exhibits a consistent pattern for SCTC across different datasets. Additionally, the SCTC results show negligible variation among different samples from the same dataset. These findings demonstrate the stability of the SCTC method compared to CytoTRACE in handling dropout events.

In single-cell studies, researchers often utilize imputation methods to address the dropout issue, which involves estimating missing gene expression values to more accurately reconstruct single-cell data (45,46). To compare the influence of imputation on pseudotime inference for CytoTRACE and SCTC methods, we selected the MAGIC imputation algorithm (45) to apply to four datasets. Specifically, we applied the MAGIC algorithm for imputation on the original datasets without prior dropout simulation, and on datasets with dropout events simulated at dropout rates of 0.1, 0.2 and 0.3, respectively.

The top row of Figure 8B displays the results of CytoTRACE across four datasets. We observed considerable variability in the impact of imputation on CytoTRACE pseudotime inference across different datasets. Overall, imputation significantly decreased the accuracy of CytoTRACE pseudotime inference. Notably, for ZEB, HSG and MSG datasets, the inferred pseudotime exhibited a negative correlation with true time points after imputation. These findings suggest that imputation methods are dataset-specific and may occasionally hinder downstream analysis performance, aligning with previous research (46).

As shown in the bottom row of Figure 8B, the SCTC method demonstrates minimal sensitivity to imputation compared to CytoTRACE. With the exception of the original HND dataset (dropout rate = 0.0), where imputation actually improved SCTC’s performance, its impact on CCI pseudotime inference is negligible in other cases. This resilience may stem from the SCTC’s ability to incorporate a wide range of network topological information, rather than relying solely on local details from individual nodes (such as gene expression levels). This feature enables SCTC to extract relatively universal and invariant characteristics from scRNA-seq data, demonstrating notable resilience to interference and robust stability.

## Discussion

The emergence of single-cell RNA sequencing at an unprecedented level of resolution presents both new opportunities and challenges in understanding complex biological processes. In this work, we introduce the concept of single-cell transcriptional complexity to infer pseudotime trajectories and developmental potential. Our findings demonstrate that this novel metric of complexity can effectively capture intricate developmental processes like neurogenesis and spermatogenesis. Our approach is inspired by the economic complexity theory, which has been successfully applied to evaluate countries’ development levels and potential. Transplanting complexity theory to the cellular context may offer fresh insights into the understanding of cellular developmental processes.

Our investigation demonstrates that during early developmental stages, while cells may exhibit relatively low gene expression levels compared to later stages, their complexity at appropriate orders of *N* is significantly high. This discovery unveils the complexity as a more robust indicator of a cell’s developmental potential than mere diversity. The underlying principle is that the coordinated interaction of pluripotency genes within highly interconnected networks, rather than the expression intensity of genes associated with pluripotency, shapes the cellular developmental potential (47). This intricate aspect can be quantified through our complexity measures.

Building upon transcriptional complexity, we have developed an unsupervised and efficient approach for single-cell pseudotime inference. Our model relies solely on the count matrix, without needing to select the highly variable genes (29). The pseudotime trajectory obtained through our complexity-based method exhibits a more accurate alignment with the actual temporal labels of cells compared to the diversity-based CytoTRACE (Figure 2). This improvement is particularly notable in the earliest developmental stages, where cells may express fewer genes than in later stages. This discrepancy can be attributed to the fact that CytoTRACE’s fundamental assumption based on gene diversity does not hold true under these circumstances.

In addition to CytoTRACE, the dedicated computational tools have recently emerged for inferring single-cell developmental potential, such as CCAT (48) and FitDevo (49). While these supervised methods have surpassed CytoTRACE on some datasets, their reliance on prior knowledge or training data may limit their generalization ability. As demonstrated in Supplementary Figure S12, our unsupervised SCTC method performs comparably to FitDevo on its training data (HND) and significantly outperforms FitDevo on non-training datasets (ZEB, HSG, and MSG). Notably, FitDevo shows inaccurate early-stage inference in ZEB data, similar to CytoTRACE. This is because zebrafish was excluded from FitDevo’s training set due to the limited homology with mammals (49). Therefore, our unsupervised complexity approach may be better suitable for such uncovered datasets, underscoring the importance of techniques like SCTC in scenarios lacking reliable training data.

Moreover, the robustness of our method extends to heterogeneous datasets, which is evident in its successful application to mixed data. This resilience to dataset heterogeneity and the transferability of our method across datasets enable cross-species exploration of cellular and gene complexity. Additionally, the stability and robustness of SCTC methods to dropout and dropout imputation imply that SCTC metrics capture relatively universal and invariant information within scRNA-seq data.

Our approach integrates the concept of economic complexity into the field of single-cell analysis, yielding meaningful insights that suggest certain inherent similarities between biology and economic complex systems. Furthermore, the breadth of theories and techniques covered within the field of economic complexity (20,34,50,51) offers an exciting avenue to extend these methods to single-cell studies and beyond. This cross-disciplinary exchange may offer new insights into understanding biological development from the perspective of complex systems (52,53).

## Supplementary Material

gkae340_Supplemental_File

## Data Availability

The source code and the data of filtered human neuron differentiation (HND) are available at https://github.com/hailinphysics/sctc, and at the Zenodo repository (https://doi.org/10.5281/zenodo.10777275). The raw data of HND (17) can be accessed from Gene Expression Omnibus (GEO) through the accession number GSE102066. Zebrafish embryonic cells (ZEB) (22) dataset can be accessed from GEO under accession number GSE112294. Human and macaque spermatogenesis datasets (23) are available under the GEO accession number GSE142585.
