# Supplementary material for: SCTC: inference of developmental potential from single-cell transcriptional complexity
Source: Nucleic Acids Res. 2024 May 6;52(11):6114–28. doi: 10.1093/nar/gkae340 (PMC11194082; doi:10.1093/nar/gkae340)
Supplement: gkae340_Supplemental_File [file gkae340_supplemental_file.pdf]

Supplementary Figure S1

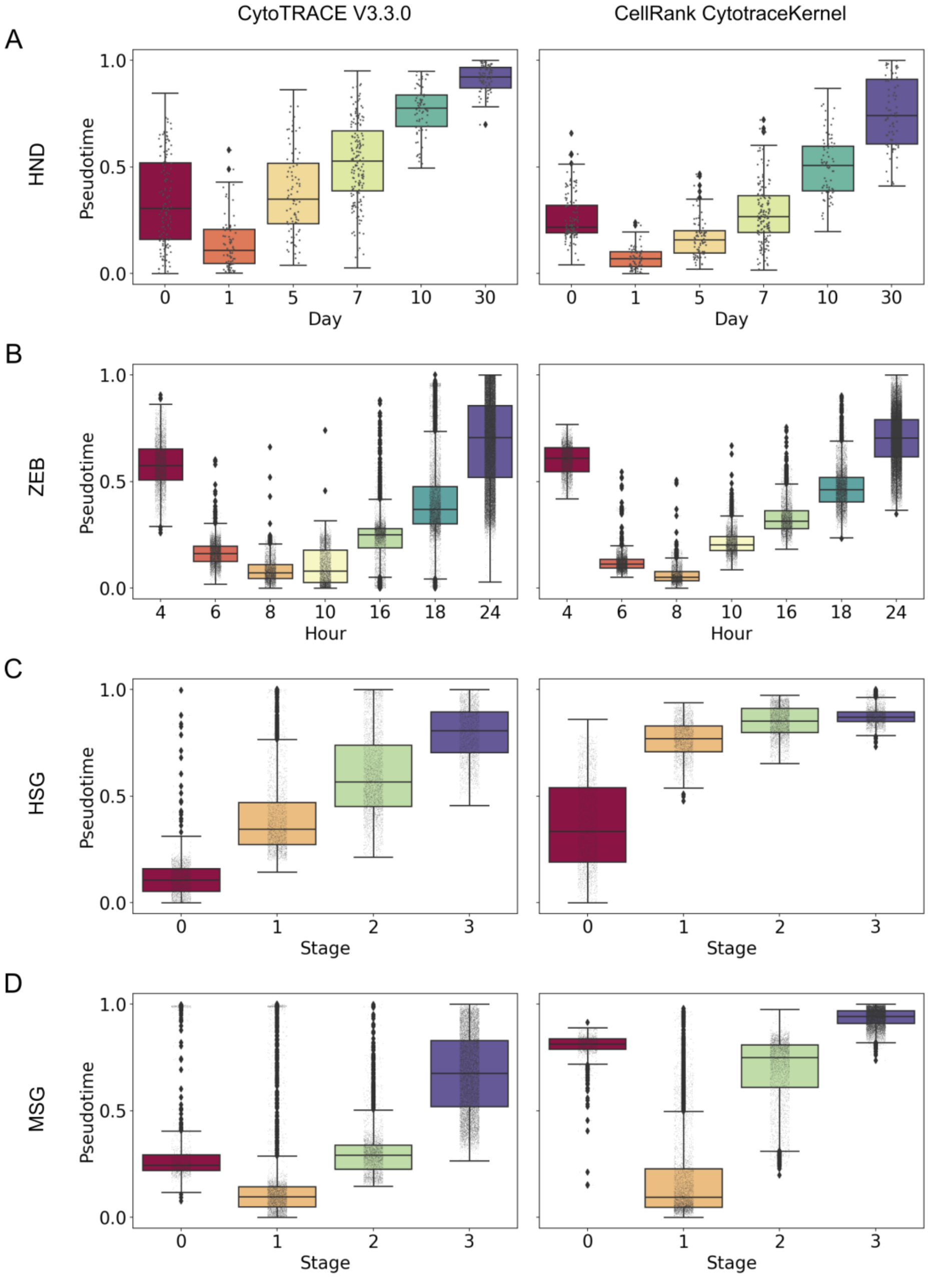

Comparison of the pseudotime calculated by CytoTRACE R package v0.3.3 and by the "CytotraceKernel" function of CellRank

The pseudotime results obtained from both CytoTRACE R package (left column) and CellRank (right column) are presented for (A) HND data, (B) ZEB data, (C) HSG data, and (D) MSG data. Although there are some differences between the two methods, It's worth noting that these discrepancies do not impact the conclusions drawn in the article.

Supplementary Figure S2

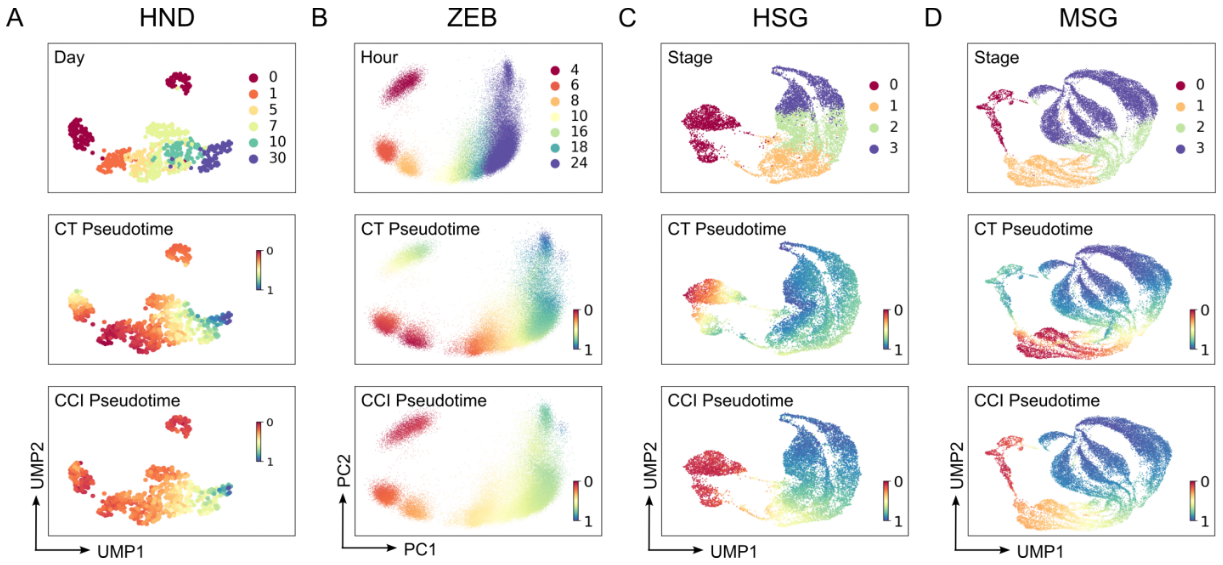

Comparison of CytoTRACE (CT) and CCI pseudotime

(A) HND data, (B) ZEB data, (C) HSG data, and (D) MSG data. Each dataset is shown using UMAP or PCA plots of time point labels, CT pseudotime, and CCI pseudotime. Compared to the CT pseudotime based on SCTD, the CCI pseudotime inferred from SCTC provides a more accurate reflection of the temporal ordering of cells during development, particularly in the early stages.

Supplementary Figure S3

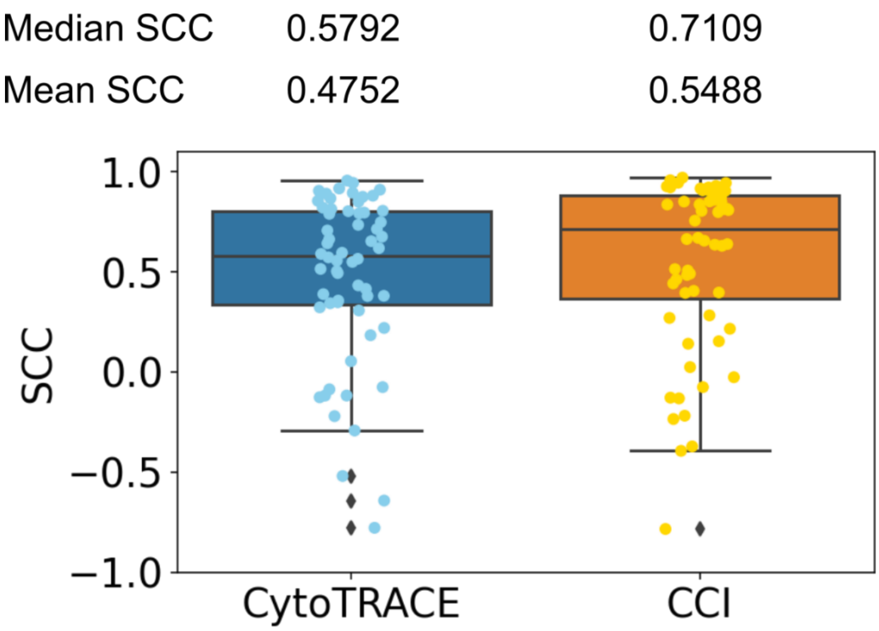

Comparison of CytoTRACE and SCTC Methods on 60 scRNA-seq Datasets

Boxplots depict the distribution of Spearman correlation coefficient (SCC) values across 60 datasets (Table S1) to compare the performance of CytoTRACE and SCTC methods. SCTC outperformed CytoTRACE in 39 (65%) datasets (Wilcoxon rank-sum test P-value = 0.035).

Supplementary Figure S4

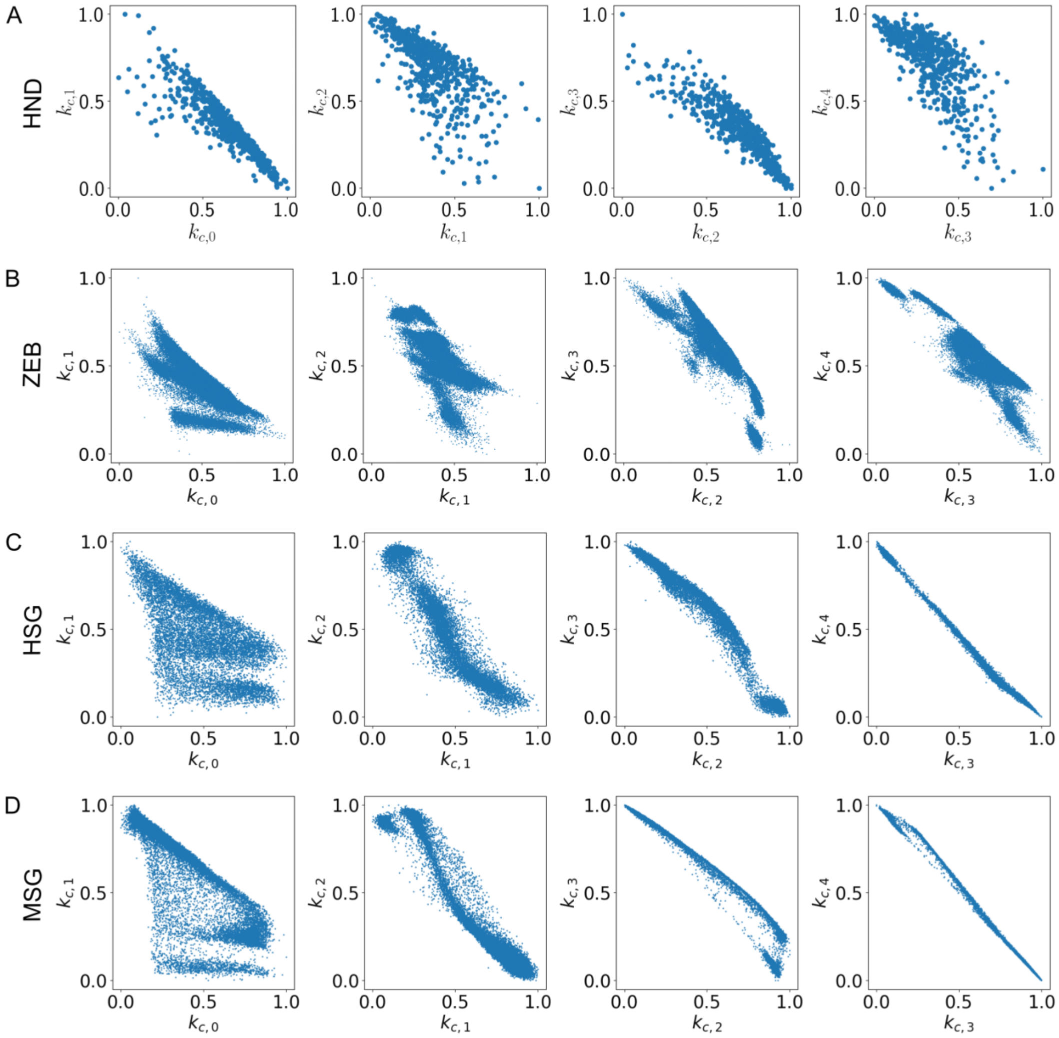

Negative Correlation between Odd-Order and Even-Order Cell Complexities

The plots illustrate the differences between the odd-order and even-order complexities ($k_{c,0}$-$k_{c,1}$, $k_{c,1}$-$k_{c,2}$, $k_{c,2}$-$k_{c,3}$, and $k_{c,3}$-$k_{c,4}$) for (A) HND data, (B) ZEB data, (C) HSG data, and (D) MSG data. The negative correlation between odd-order and even-order complexities is evident across four datasets.

Supplementary Figure S5

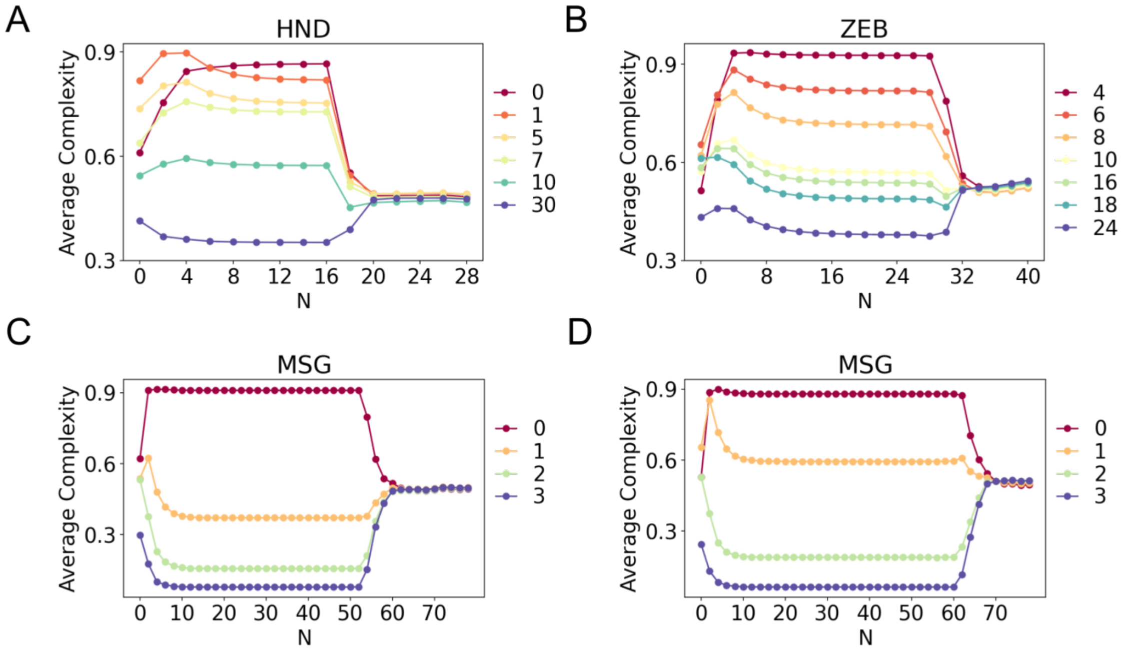

Threshold Order for Complexity Convergence

Recursion will cause the cell complexity to collapse to the same value when the complexity order *N* exceeds a certain threshold N_{th}. The threshold order N_{th} at which this collapsing occurs is determined for each dataset: (A) N_{th} = 16 for HND data, (B) N_{th} = 28 for ZEB data, (C) N_{th} = 52 for HSG data, and (D) N_{th} = 60 for MSG data, respectively.

Supplementary Figure S6

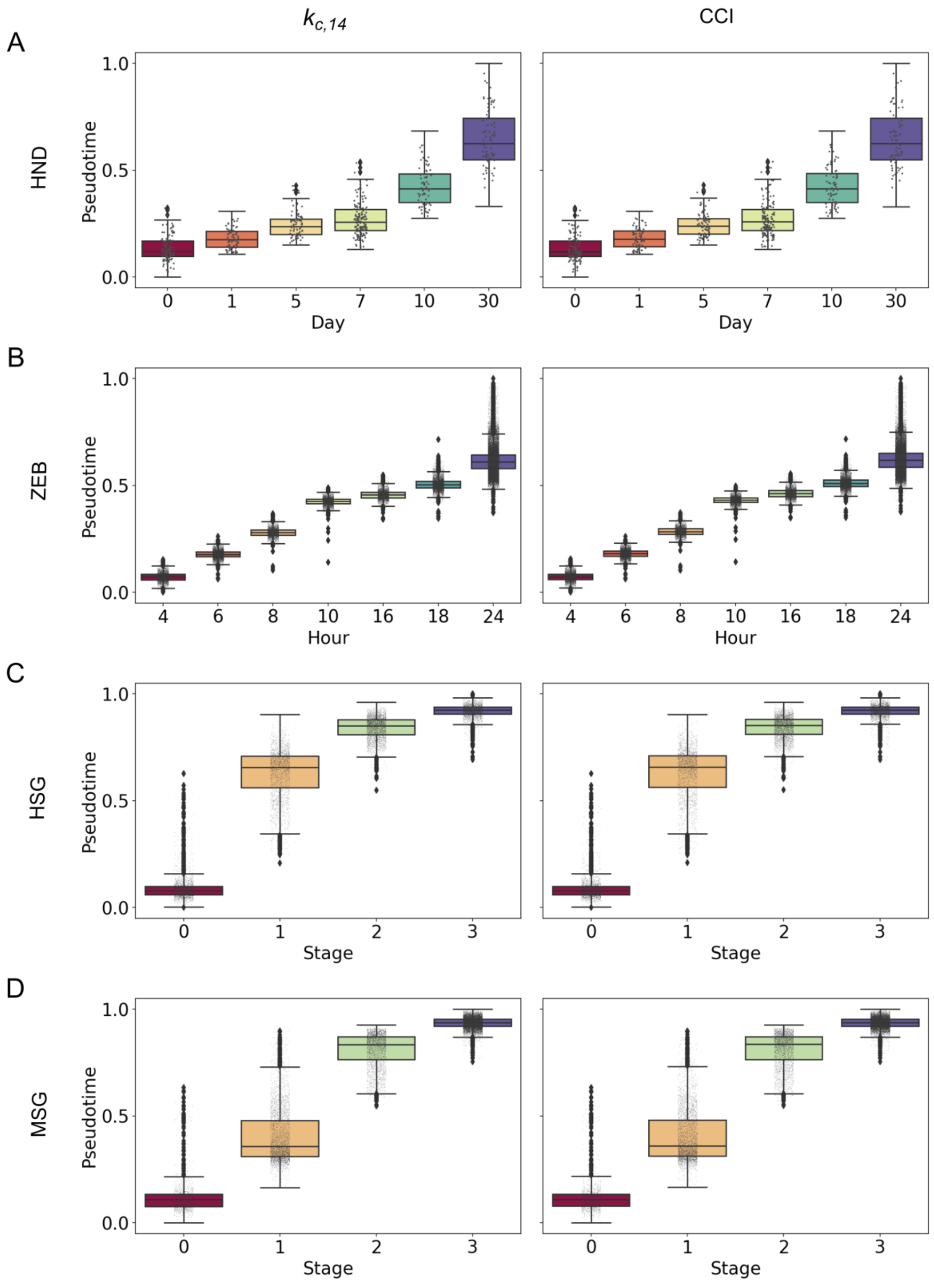

Comparison of Pseudotime Inference between $k_{c,\,14}$ and CCI

Pseudotime inferred using 14-order cell complexity ($k_{c,14}$, left column) versus Cell Complexity Index (CCI, right column) on (A) HND, (B) ZEB, (C) HSG, and (D) MSG scRNA-seq datasets. The pseudotime orderings obtained by the two methods show high concordance across all four datasets, indicating $k_{c,14}$ provides equivalent pseudotime inference to CCI.

Supplementary Figure S7

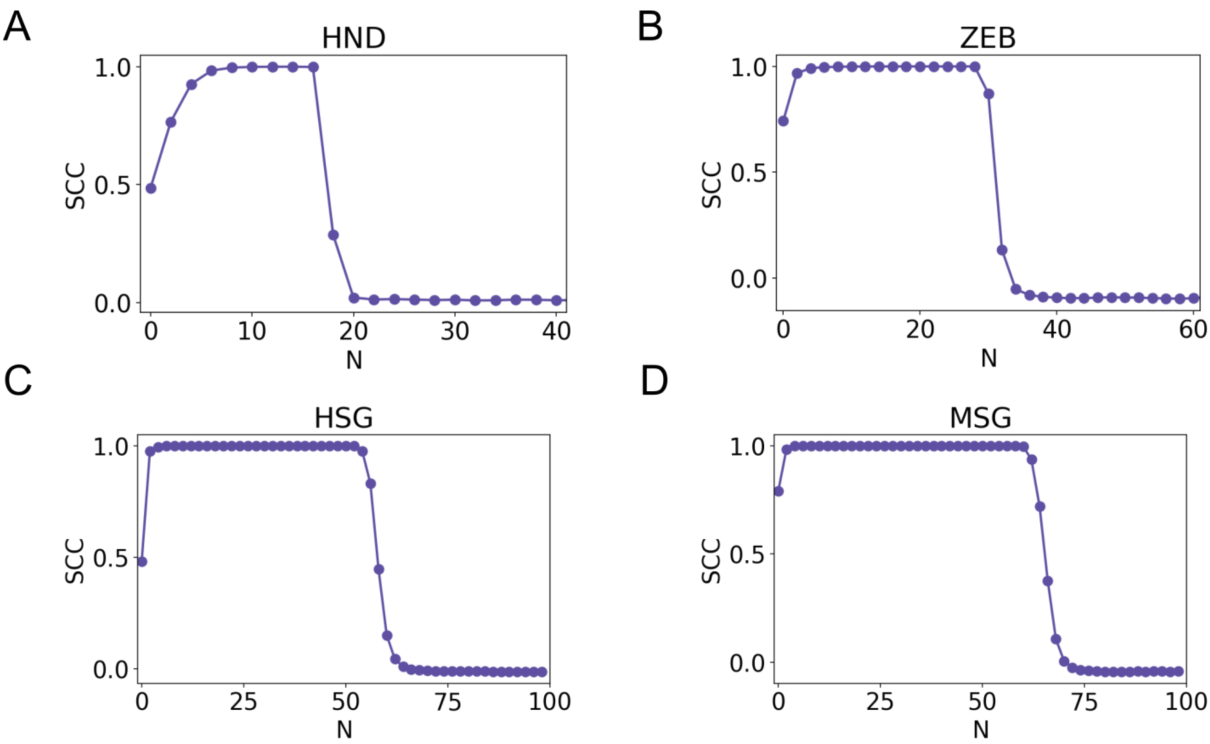

Correlation Between *N*th-Order Cell Complexity and Cell Complexity Index (CCI)

We present the Spearman Correlation Coefficients (SCCs) between various *N*th-order complexities and CCI across four datasets: (A) HND, (B) ZEB, (C) HSG, and (D) MSG. The figure illustrates the consistency between CCI and the *N*th-order complexity within a certain range of *N* values. When utilizing the *N*th-order complexity, it's critical to identify an appropriate *N* value. During the iterative computation of the *N*th-order complexity, the calculation can be stopped when the SCC value between the *N*th-order complexity and CCI is sufficiently high (approaching 1), with the current *N* value being used as the criterion. Alternatively, a broader range of *N* values can be explored for *N*th-order complexity, and the *N* value exhibiting the highest correlation with CCI can be chosen as the criterion. Specifically, the *N* values corresponding to the maximum SCC for the four datasets are as follows: 14 for HND, 24 for ZEB, 34 for MSG, and 30 for HSG.

Supplementary Figure S8

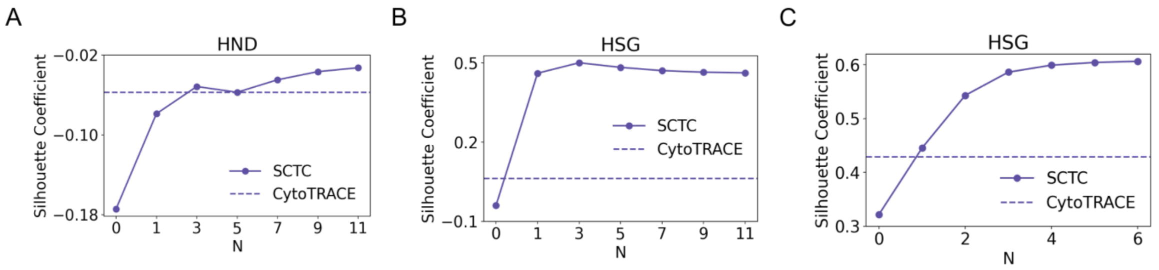

Silhouette Coefficients of Gene Complexity across Complexity Orders

Silhouette coefficients measuring the consistency of gene complexity with developmental time at different complexity orders *N*, calculated based on scRNA-seq datasets: (A) HND, (B) HSG, and (C) MSG. Overall, the silhouette coefficient increases with higher complexity order N, indicating that higher-order complexities provide better distinction of developmental stages.

Supplementary Figure S9

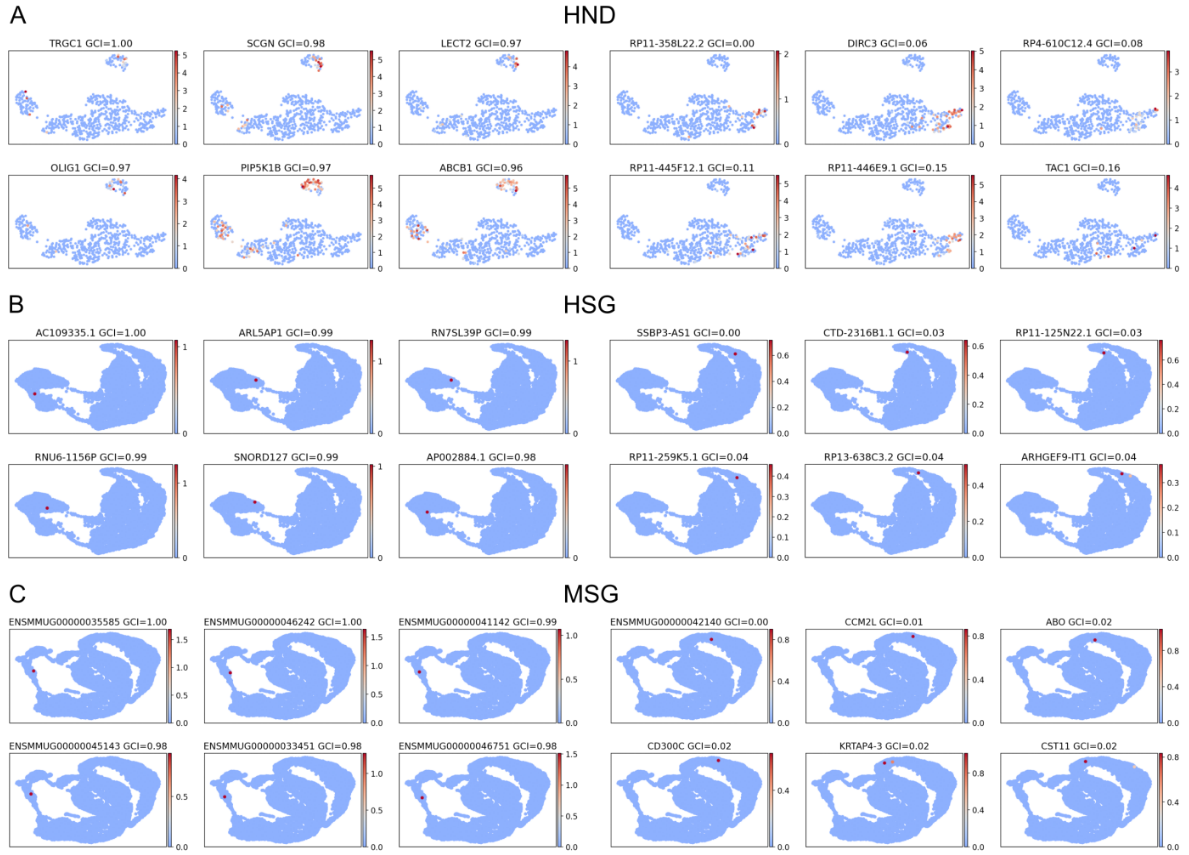

Expression Distributions of Top 6 Genes with Highest and Lowest Complexity
The expression distributions of the highest 6 complexity genes (left column) and the lowest 6 complexity genes (right column) are shown for (A) HND, (B) HSG, and (C) MSG scRNA-seq datasets. In each dataset, genes with the highest complexity predominantly exhibit expression at the initial time point of development, while genes with the lowest complexity are primarily observed at the final time point.

Supplementary Figure S10

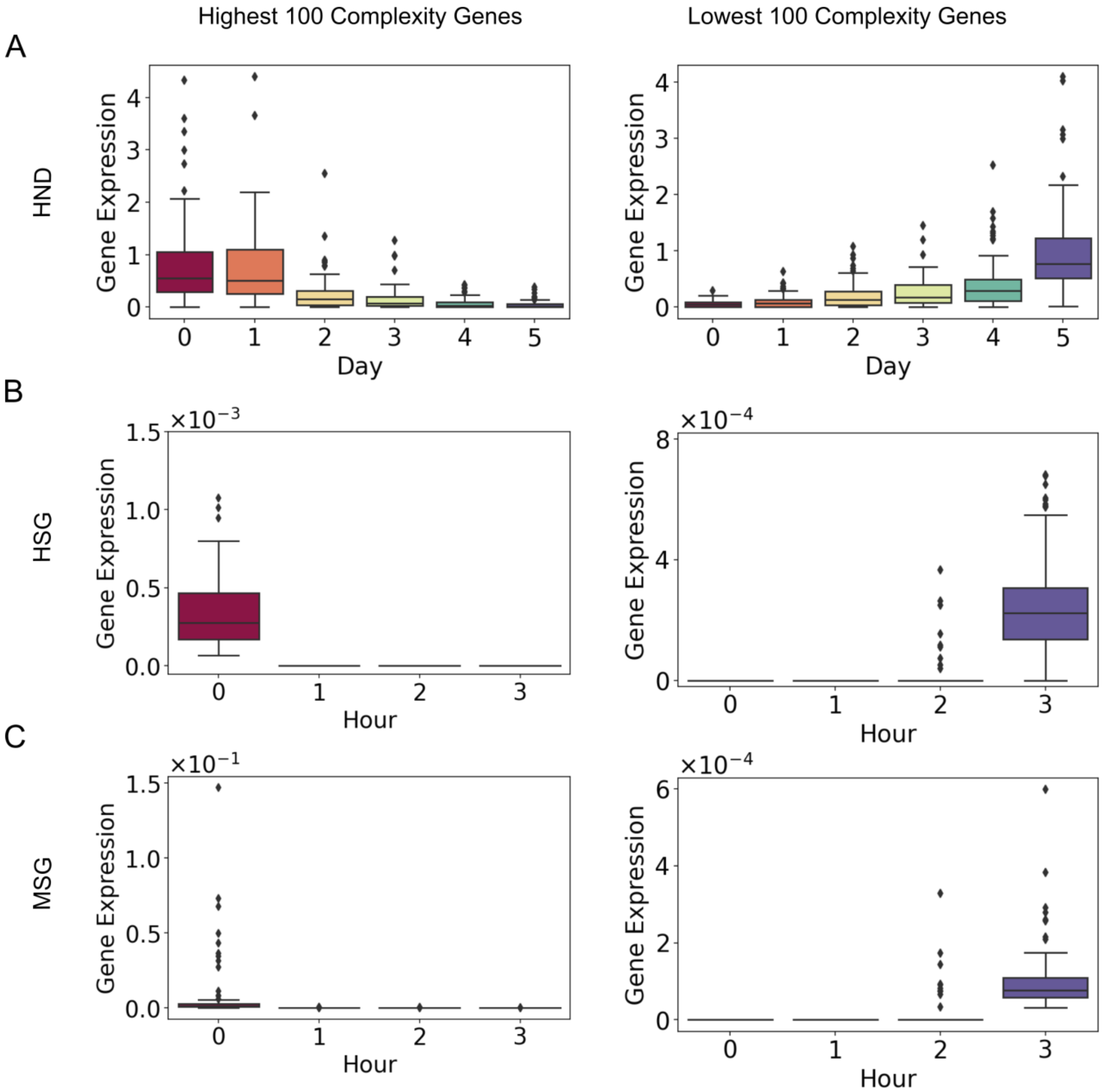

Expression Distributions of the Top 100 Genes with Highest GCI and Lowest GCI
The expression distributions of the top 100 genes with the highest GCI (left column) and the lowest GCI (right column) for (A) HND data, (B) HSG data, and (C) MSG data. Across all datasets, genes with high complexity are preferentially expressed in early development, while genes with low complexity exhibit specificity for later stages.

Supplementary Figure S11

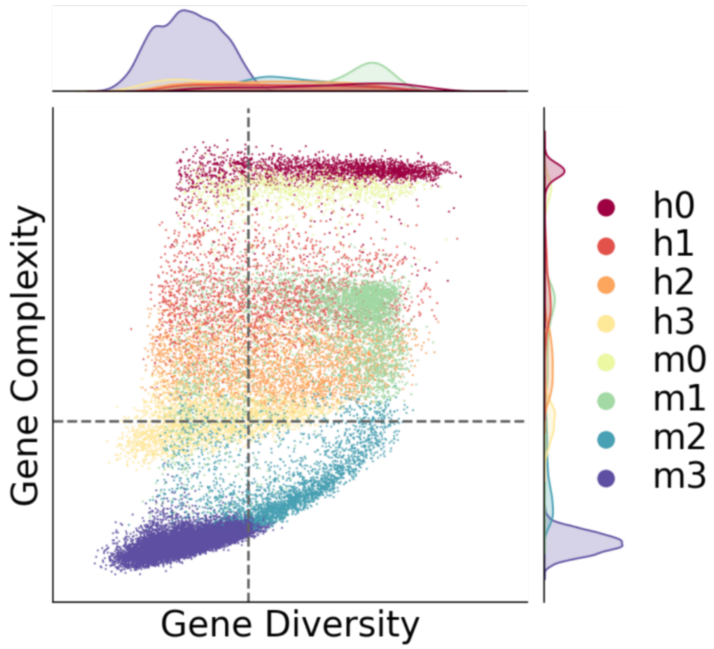

Diversity-Complexity Analysis of Mixed HSG and MSG Dataset
The diversity-complexity diagrams illustrate the single-cell gene expression profiles for the mixed HSG and MSG dataset, where 'h' represents human samples, 'm' represents macaque samples, and digits denote different developmental stages. The analysis shows that the gene diversity between human and macaque sperm cells exhibits minimal variation, but the gene complexity of human sperm cells is notably higher overall compared to macaque sperm cells.

Supplementary Figure S12

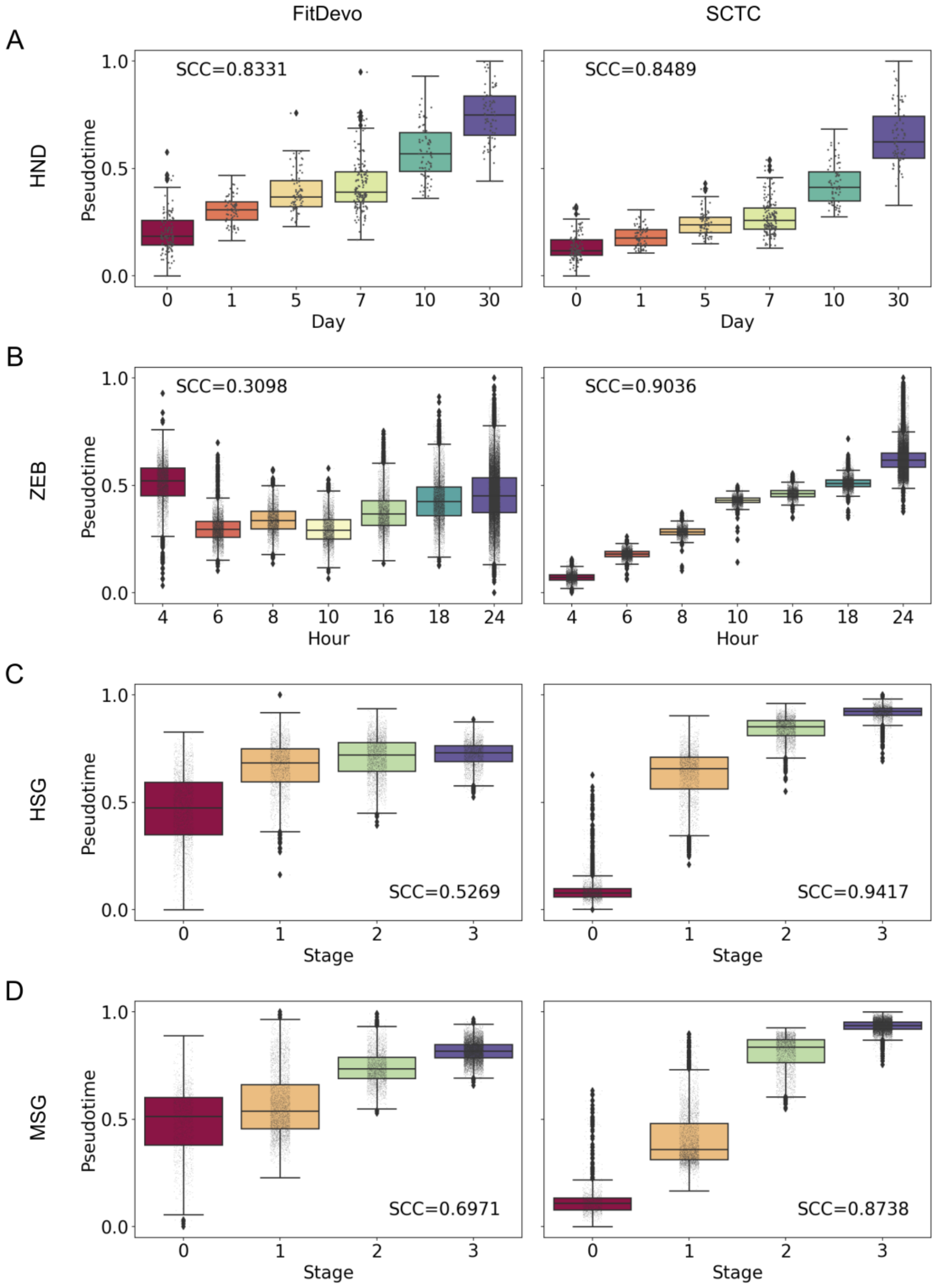

Comparison of Pseudotime Inference Between FitDevo and SCTC

The pseudotime inference results obtained by FitDevo (left column) and SCTC (right column) are compared. For the HND dataset (A), which is one of the training datasets for FitDevo, both methods demonstrate a high Spearman Correlation Coefficient (SCC). However, for datasets not used for FitDevo training, including (B) ZEB, (C) HSG, and (D) MSG, SCTC as an unsupervised method significantly outperforms FitDevo.

Supplementary Table S1: Evaluating the Performance of SCTD and SCTC on 60 Datasets

Index	Dataset	CytoTRACE SCC	CCI SCC
1	Aging HSCs (Smart-seq2)	-0.5204	**-0.3729**
2	Blastocyst phenotypes (SC3-seq)	0.8030	**0.8490**
3	Blastocyst timepoints (SC3-seq)	0.3776	**0.8117**
4	Cortical interneurons (C1)	0.4307	**0.4892**
5	Dentate gyrus phenotypes (10x)	**0.6407**	-0.2196
6	Dentate gyrus timepoints (10x)	**0.7048**	-0.0770
7	Embryonic HSCs (Tang et al.)	**0.5129**	0.5120
8	Endometrium (CEL-seq)	0.7116	**0.8093**
9	Peripheral glia (Smart-seq2)	0.8482	**0.9199**
10	Medial ganglionic eminence (C1)	**0.4134**	-0.0275
11	Dendritic cells (C1)	0.8527	**0.8842**
12	Hair epidermis (C1)	-0.6429	**-0.1332**
13	HSPCs (C1)	**-0.7788**	-0.7847
14	Hepatoblast (Smart-seq2)	**0.8115**	0.6282
15	HSMM (C1)	0.6611	**0.8579**
16	Mesoderm (C1)	0.7874	**0.8754**
17	Peripheral blood (10x)	**0.4922**	0.3952
18	Pre-implant human embryo (Tang et al.)	0.9422	**0.9555**
19	Germ cells (Smart-seq2)	-0.1190	**0.0229**
20	mESC in vitro (RamDA-seq)	**0.5015**	0.4405
21	Lung development (C1)	0.7904	**0.9025**
22	Direct in vitro neuron (inDrop)	0.8884	**0.9192**
23	Lgr5-CreER intestine (CEL-seq)	**0.5545**	0.1345
24	Bone marrow (10x)	**0.5935**	0.2683
25	Bone marrow (Smart-seq2)	**0.8011**	0.6619
26	Intestine (Smart-seq2)	**0.6514**	0.5049
27	Standard in vitro neuron (inDrop)	0.8650	**0.9275**
28	Pre-implant mouse embryo	0.5479	**0.8498**
29	Lung fibroblast (C1)	0.5633	**0.8132**
30	Intestine (Drop-seq)	0.3213	**0.6678**
31	Neural stem cells (Drop-seq)	**0.3056**	-0.2361
32	Skeletal stem cells (C1)	0.5706	**0.6340**
33	In vitro NPCs (C1)	0.5877	**0.7964**
34	Oligodendrocyte phenotypes (C1)	-0.0769	**0.6537**
35	Oligodendrocyte timepoints (C1)	**0.3787**	0.1520
36	Pancreatic beta cell (Smart-seq2)	**0.8784**	0.8344
37	Whole planaria (Drop-seq)	0.3465	**0.8022**
38	Thymus (Drop-seq)	0.3871	**0.7540**

39	Early zebrafish (Drop-seq)	0.7938	**0.9249**
40	developing-dendritic-cells_schlitzer	0.9081	**0.9141**
41	human-embryos_petropoulos	-0.2217	**0.8065**
42	mESC-differentiation_hayashi	0.8726	**0.9683**
43	myoblast-differentiation_trapnell	0.3550	**0.4035**
44	pancreatic-alpha-cell-maturation_zhang	0.8923	**0.8960**
45	psc-astrocyte-maturation-glia_sloan	**-0.2933**	-3948
46	psc-astrocyte-maturation-neuron_sloan	**-0.0880**	-0.1296
47	stimulated-dendritic-cells-LPS_shalek	0.2184	**0.3931**
48	stimulated-dendritic-cells-PAM_shalek	0.1820	**0.4603**
49	stimulated-dendritic-cells-PIC_shalek	**0.6739**	0.2143
50	farrell_2018	**0.9146**	0.9052
51	briggs_2018 (GSE113074)	-0.1275	**0.6368**
52	chu1_GSE75748_sc_cell_type_ec	**0.9538**	0.9395
53	chu2_GSE75748_sc_time_course_ec	-0.1191	**0.8341**
54	Trapnell GSE52529_fpkm_matrix	0.0526	**0.2812**
55	Yao_GSE86977_UMI_20K.2684	**0.9027**	0.4828
56	Mouse Spermatogenesis	0.3418	**0.9429**
57	Human Neuron Differentiation (HND)	0.6166	**0.8489**
58	Zebrafish Embryonic Cells (ZEB)	0.7327	**0.9036**
59	Human Spermatogenesis (HSG)	0.7461	**0.9417**
60	Macaque Spermatogenesis (MSG)	0.8186	**0.8738**

Notes:

- Datasets 1-39 were selected from the 42 benchmark datasets provided by CytoTRACE (13). We excluded the 30th dataset (AT2/AT1 lineage (C1)) because we found that the time labels did not match the actual developmental stages. Additionally, we removed two datasets that overlapped with the QuasiIdr benchmark datasets (30).
- Datasets 40-51 were obtained from the QuasiIdr benchmark datasets (30), which primarily derive from the work of Saelens et al. (12), with the addition of a few extra datasets. The QuasiIdr benchmark datasets contain three types of standards: gold, silver, and other. We selected 12 gold-standard datasets with a linear trajectory type and two other-standard datasets (dataset 50 and dataset 51) from model organismal single-cell developmental studies.
- While dataset 51 (briggs_2018) originates from the QuasiIdr benchmark datasets, its preprocessed format does not meet CytoTRACE's input requirements. Therefore, we alternatively obtained the raw data from Gene Expression Omnibus (GEO) using accession number GSE113074 (33).
- Datasets 52-55 were retrieved from the scRNA-seq datasets used in NCG method (31).

- Dataset 56 (Mouse Spermatogenesis), similar to HSG and MSG datasets, is one of the mammalian spermatogenesis scRNA-seq datasets published by Shami et al (23, 32).
- For duplicate datasets from different sources, we retained only one with the highest average Spearman Correlation Coefficient (SCC) calculated using both the SCTD and SCTC methods.
- The datasets sourced from the CytoTRACE benchmark datasets have undergone normalization and log1p transformation, so we did not perform any additional preprocessing on them. However, the datasets from Quasildr and NCG were only normalized, so we proceeded to apply log1p transformation to those datasets. All other data underwent both normalization and log1p preprocessing.
